# Environmental Predictors of Diversity in Recent Planktonic Foraminifera as Recorded in Marine Sediments

**DOI:** 10.1371/journal.pone.0165522

**Published:** 2016-11-16

**Authors:** Isabel S. Fenton, Paul N. Pearson, Tom Dunkley Jones, Andy Purvis

**Affiliations:** 1 Department of Life Sciences, Natural History Museum, Cromwell Road, London SW7 5BD, United Kingdom; 2 Department of Life Sciences, Imperial College London, Silwood Park Campus, Ascot SL5 7PY, United Kingdom; 3 School of Earth and Ocean Sciences, Cardiff University, Cardiff CF10 3AT, United Kingdom; 4 School of Geography, Earth and Environmental Sciences, University of Birmingham, Edgbaston, Birmingham B15 2TT, United Kingdom; Union College, UNITED STATES

## Abstract

Global diversity patterns are thought to result from a combination of environmental and historical factors. This study tests the set of ecological and evolutionary hypotheses proposed to explain the global variation in present-day coretop diversity in the macroperforate planktonic foraminifera, a clade with an exceptional fossil record. Within this group, marine surface sediment assemblages are thought to represent an accurate, although centennial to millennial time-averaged, representation of recent diversity patterns. Environmental variables chosen to capture ocean temperature, structure, productivity and seasonality were used to model a range of diversity measures across the world’s oceans. Spatial autoregressive models showed that the same broad suite of environmental variables were important in shaping each of the four largely independent diversity measures (rarefied species richness, Simpson’s evenness, functional richness and mean evolutionary age). Sea-surface temperature explains the largest portion of diversity in all four diversity measures, but not in the way predicted by the metabolic theory of ecology. Vertical structure could be linked to increased diversity through the strength of stratification, but not through the depth of the mixed layer. There is limited evidence that seasonal turnover explains diversity patterns. There is evidence for functional redundancy in the low-latitude sites. The evolutionary mechanism of deep-time stability finds mixed support whilst there is relatively little evidence for an out-of-the-tropics model. These results suggest the diversity patterns of planktonic foraminifera cannot be explained by any one environmental variable or proposed mechanism, but instead reflect multiple processes acting in concert.

## Introduction

Present-day macroecological patterns result from a combination of current environmental and longer-term historical factors acting on clades (e.g. [1, 2]). Typically, past and present causes are considered separately: macroecologists usually relate spatial patterns in Recent diversity to current environments [3, 4], whereas palaeobiologists seek historical causes for spatial and temporal patterns in fossil diversity [5, 6]. The disconnect between these two approaches has hampered development of an integrated understanding of these macroecological patterns [7], exacerbated by a discipline-specific focus on different groups. Most neo-macroecological studies concentrate on charismatic terrestrial groups with good distribution records, such as birds (e.g. [4]); unfortunately these groups often have relatively poor fossil records. Conversely, groups with excellent fossil records are usually marine and are less well-known in modern environments [8].

As a step towards bridging this divide between neo- and palaeo-macroecology, we present a new global analysis of Recent site-level diversity of macroperforate planktonic foraminifera—the group with the best species-level fossil record currently known [9]. We go beyond previous macroecological work on this clade (e.g. [10–13]) by considering multiple diversity measures, including functional diversity and mean evolutionary age, to help tease apart different ecological and evolutionary hypotheses.

### Planktonic foraminifera as a model system

Planktonic foraminifera are unicellular zooplankton, with a test or ‘shell’ made of calcium carbonate [14]. Upon death, these tests accumulate on the sea floor and are buried as a biogenic component of marine sediments, building up a continuous record of foraminiferal assemblages through geological time. Wide sampling of these sediments, both in the Recent (coretops) and through deep time (by the International Ocean Discovery Program and its predecessors), has produced rich data on species composition and relative abundance. Sediment samples are inevitably time-averaged, on the order of hundreds to a few thousand years [15]. Time-averaging limits temporal resolution but has the dual benefits of averaging away short-term (e.g. seasonal) fluctuations and de-emphasising recent human impacts, which can otherwise confound macroecological studies (e.g. [16]). A meta-analysis comparing life and death assemblages in shallow marine environments found that rank abundance is preserved [16], though species with shorter generations are likely to be disproportionately abundant in the sediment.

Species-level identification of planktonic foraminifera, whether extant or extinct, is based on test morphology, making modern and fossil data directly comparable. This study focuses on the macroperforate clade, whose tests are less susceptible to dissolution than those of microperforate species [14] and are easier to identify to morphospecies from the size fraction of the samples in this analysis. Thirty evolutionarily distinct extant macroperforate species can be identified from test morphology [9], although many of them harbour distinct genotypes that might be biological species [17]. These genetic lineages are mostly geographically separated [18], so will influence alpha diversity estimates much less than global diversity. Aze et al. [9] published a complete phylogeny of the clade for the Cenozoic (last 66 Ma) tracing fossil relationships based solely on morphology.

### Facets of diversity

**Fig 1:**
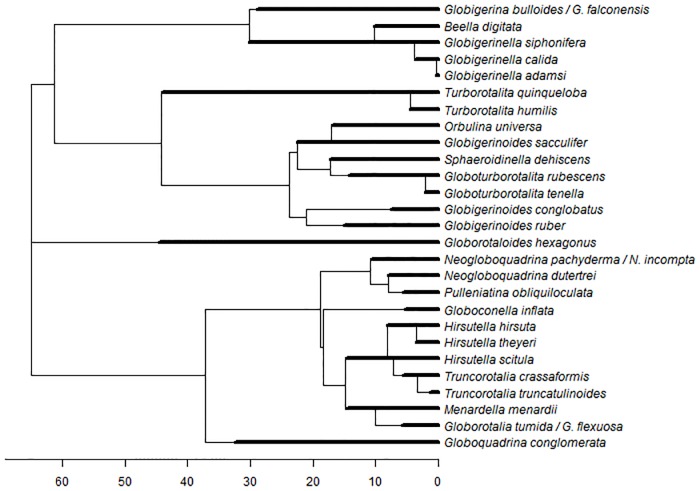
Lineage phylogeny of the macroperforate planktonic foraminifera based on Aze et al. [9]. This phylogeny contains all the lineages present in this study scaled by age. The axis shows the node age in million years (Ma). The thicker lines represent the lineage ages. The lineage ages do not always map directly onto the phylogeny. The phylogeny plots each speciation event as the origination of two new species; however some lineages persist through speciation events, so some of the age lines extend beyond the node. Some ages are also shorter than the branch; this is the result of speciation events that produced species that are now extinct. Some morphological species are part of the same evolutionary lineage.

The most widely-used and easily interpreted measure of assemblage-level diversity is species richness. However, no single number can adequately reflect all facets of assemblage diversity [19], so we consider three additional measures: evenness, functional diversity and mean evolutionary age. Evenness quantifies the relative abundance of species, showing how the available resources are apportioned [20]. Functional diversity reflects the value and range of functional traits of the organisms present in a given ecosystem [21]. Mean evolutionary age (MEA) is the (abundance-weighted) average age of the species lineages present in an assemblage. The exceptional fossil record of this clade [9] means ages can be estimated much more meaningfully than is possible from molecular phylogenies, where the usual measure—terminal branch length—is influenced by the longevity of other species and does not consider persistence of a lineage through speciation [22]. Species’ ages could be taken to be the first occurrence of the Recent morphospecies. However, because many morphospecies apparently arise from pseudospeciation events, i.e. through anagenetic rather than cladogenetic changes [9, 23], we instead use the ages of evolutionary species (from [9]) in our estimation of MEA (Fig 1). A younger MEA implies that the species in that assemblage have arisen more recently, whereas an older age implies the species have persisted for longer. Note that a community’s MEA does not imply that it has existed for that length of time, or that any speciation occurred *in situ*.

### Diversity in planktonic foraminifera

Previous diversity analyses in this group have only considered species richness (SR), limiting their ability to discriminate among competing hypotheses, many of which also make predictions about other aspects of diversity. Most previous analyses also focussed on the Atlantic, whose oceanography produces strong correlations among environmental variables, hampering efforts to statistically separate their effects. These correlations are weaker in the Indian and Pacific Oceans [24].

Rutherford et al. [10] reported that coretop planktonic foraminifera species richness peaks at latitudes around ±20°, and that a single measure of present-day environment—sea surface temperature (SST)–explains 90% of the variation in richness in the Atlantic. They suggested SST influences diversity by controlling the vertical partitioning of the water column, with warmer surface water providing more distinct niches, but did not test this idea. Brayard et al. [11] explained the shape of the Atlantic planktonic foraminiferal latitudinal diversity gradient (LDG) as a mid-domain effect (MDE: [25]) acting on the latitudinal gradient in SST (for an explanation of the MDE see the section below). More recent simulations used pseudospecies with given temperature tolerances to investigate how an MDE could interact with the SST [26]. Morey et al. [13] correlated a range of environmental variables with the observed planktonic foraminiferal community composition. Their analysis tested foraminiferal assemblages as proxies for past environmental conditions, and did not consider theoretical explanations for these correlations. Tittensor et al. [12] considered drivers of species richness in a range of marine taxa including the planktonic foraminifera. They identified SST as the main driver of diversity, with oxygen stress also being important.

**Fig 2:**
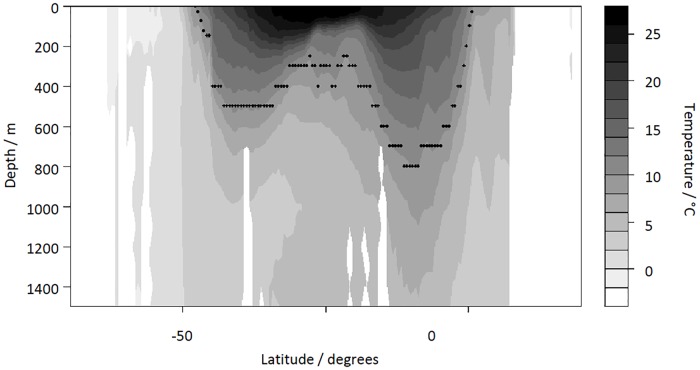
Vertical thermal structure of the ocean. A section through the Atlantic (at -33.5° longitude) showing how the thermal structure changes with latitude, measured in °C. The points highlight the 10°C depth contour.

Here we use site-level data on species richness, species evenness, mean evolutionary age and functional diversity to test the hypotheses suggested in previous planktonic foraminiferal richness studies [10–13, 26–29]. These hypotheses can be split into four broad categories (e.g. [30]) in an attempt to understand the mechanisms driving the observed correlations:

1. **Ecological limits.** The observed diversity in each site could result from having reached a fundamental ecological limit [12]. The vertical structure of communities and any seasonal variation in these are collapsed in each site, so variation in diversity could be driven by superposition of multiple assemblages (a/b). Alternatively it could result from higher diversity within given communities (c-e).
  - **Vertical temperature structure.** Rutherford et al. [10] found the most significant correlate of richness was temperature, which they linked to the creation of vertical niches. The shape of the temperature gradient (Fig 2) determines the potential for the water column to support multiple vertically-stacked communities [31]. Sites with a greater vertical temperature gradient can have multiple communities superposed, permitting higher richness and higher functional diversity. This superposition would also increase evenness, other things being equal [32].
  - **Seasonal assemblages.** Seasonally-varying environmental conditions (e.g. temperature, salinity; see below for discussion of productivity) present challenges to non-motile species to which there are two responses: either different communities occupy the same ocean region in different seasons [13, 33], or communities are dominated by eurytopic species. Seasonal communities yield the same predictions as vertical temperature structure (i.e. higher richness and functional diversity, and more even assemblages). Eurytopic species have broad environmental tolerances; if they dominate, communities should have low richness and evenness, and be less functionally diverse [34].
  - **Sea Surface Temperature**. If the richness-temperature association results from an MDE acting on the SST gradient [11, 26], then highest richness is expected at mid temperatures, while functional diversity and evenness are not expected to show any richness-independent variation. Brayard et al.’s [11] model fits best if the clade originated in mid to low latitudes (see hypothesis 2b, Out of the Tropics, below).
  - **Productivity** has been identified as important in many studies (e.g. [35]). Typically, highly productivity regions would have higher population densities, and therefore higher richness and functional diversity. Also, Hoffmann and Hercus [36] suggested productivity increases diversification, which should give a younger MEA. However, high ocean productivity mostly results from seasonal upwelling of nutrient-rich water [24], creating large annual fluctuations. **Seasonality of production** acts in the same way as seasonal assemblages (1b), either increasing or decreasing diversity.
  - **Stress.** Species living outside their optimum conditions will experience stress. For some environmental conditions, e.g. temperature, stress levels will depend on species-specific adaptions. Macroperforate planktonic foraminifera are mostly intolerant of low **oxygen** levels and very high or low **salinity** [12, 17], so we use these variables as indicators of environmental stress. Regions of high stress are predicted to have lower richness and functional diversity. Evenness would be low if a small number of species dominates. Stress has also been linked to increased diversification [36], suggesting younger MEA.
2. **Evolutionary dynamics.** These hypotheses suggest the system is not currently at equilibrium, with diversity patterns reflecting variation in immigration, extinction and speciation.
  - **Stability in deep time** predicts geologically older environments harbour more diversity as their communities have had longer to assemble [6], a relationship observed in terrestrial and coastal systems (tropical niche conservatism: [6, 37]). As planktonic foraminifera are able to disperse rapidly [38, 39] this hypothesis would only hold if species are less able to establish in new environments (i.e. phylogenetic niche conservatism). This scenario is consistent with the diversity-dependent dynamics seen within planktonic foraminifera [40, 41] and predicts lower richness and younger MEA in younger environments.
  - **The Out-of-the-tropics model** suggests that taxa preferentially originate in the tropics before expanding their ranges outwards [42]. Many other studies have identified higher tropical speciation rates as important [27, 28]. This hypothesis predicts higher richness and younger MEA in the tropics, as younger species have not had sufficient time to expand to higher latitudes [43].
  - The **metabolic theory of ecology** (MTE) links higher temperature to higher speciation rates. This model aims to explain many ecological phenomena using simple principles [44, 45]. It predicts the temperature-dependence of richness has a slope of -0.65 when log(richness) is regressed on 1 / kT, where k is the Boltzmann constant (eV K-1) and T is the SST (K) [46]. Marine bacteria show this expected relationship [47].
3. **Statistical.** The Mid Domain Effect (MDE) arises if species are randomly placed in space in which case, statistically, species are more likely to be placed in the centre of a domain than at the edges [25]. Diversity is therefore predicted to peak towards the centre of ocean basins, in both latitude and longitude. If the domain consists of one hemisphere then a richness gradient with a peak in temperate latitudes is expected [48]. This hypothesis predicts that the patterns of richness will be basically identical across the Atlantic and Pacific oceans but differ in the Indian, as the latter does not extend far into the northern hemisphere.
4. **Dissolution** of calcite increases with depth in the water-column. Susceptibility to dissolution after death varies among planktonic foraminiferal species [49], with significant dissolution biasing the recorded core top diversity to lower values [49].

Environmental variables were chosen to reflect these hypotheses, and incorporated into global spatial analyses of multiple diversity measures.

## Methods

### Materials

#### Planktonic foraminiferal data

The MARGO database [50] contains species abundance data from the 150μm size fraction of 3773 sediment coretops across the world’s oceans. Each record has counts of intact specimens of all planktonic foraminiferal morphospecies, as well as the coordinates of the sample site. Roughly 300–500 intact specimens were counted at each site (min: 275, median: 419, max: 2664 in this dataset). Although previous studies have shown that using the 150μm size fraction tends to slightly underestimate polar and equatorial richness [31], data for the more optimal sieve-size of 125μm are much more limited. The underestimation is reduced by our focus on macroperforate species, rather than on the (typically smaller) microperforates. For a full list of the species used in this analysis see S1 Table. Sites where *Globorotalia menardii* and *G*. *tumida* have been merged were excluded from the analysis, as were sites where *Truncorotalia crassula*, an extinct species, has been recorded. *Globigerinoides ruber* has not been split into pink and white forms to avoid a paraphyletic lineage, as the MARGO database does not separate *G*. *elongatus* from *G*. *ruber* (white) [51]. Lineage ages were obtained from the phylogeny of Aze et al. [9], where some morphological species have been grouped into evolutionary lineages. Although some aspects of this taxonomy may not be ideally resolved, we considered it more important to use a methodologically uniform and internally consistent data source.

We used specimen-based rarefaction [52, 53] to produce sample-size-independent estimates of site-level species richness (SR). Hurlbert’s [52] formula was used to calculate rarefaction estimates based on 275 individuals; approximately 200 sites with fewer individuals were excluded from the analysis. The 285 sites with no count of the total individuals were excluded only from the dataset used for the richness model. The species evenness measure we used divides Simpson’s diversity by the observed richness [54], so is independent of richness [55]. Higher values indicate more equal abundances among species. As evenness measures are unbiased by sample size, rarefaction is unnecessary. As mentioned above, the MEA was calculated as the abundance-weighted mean age of species at a site. To estimate functional diversity we use Villéger et al.'s [56] measure, FRic, which calculates the amount of functional trait space occupied by the assemblage. The traits used relate to both morphological and ecological characteristics of the species (see Table 1).

**Table 1:** A summary of the traits used to calculate functional richness.

Trait	Description	Source
Spinose	Presence or absence of spines on the test	Aze et al. [9]
Structure	Grouping based on the test wall ultrastructure (Cancellate, coarsely cancellate, irregularly cancellate, cancellate with smooth cortex, hispid, smooth)	Aze et al. [9]
Dissolution	Susceptibility of the test to dissolution—another characteristic of shell structure	Berger [49]
Area	The square-root of test area taken from photographs	Veal [57]
Morphogroup	Classification based on distinctive test morphology (Spinose: flat, globular, globular with supplementary apertures, spherical, clavate, planispiral. Non-spinose: globular, globorotaliform keeled, globorotaliform anguloconical.)	Aze et al. [9]
Ecogroup	Classification based on ecological characteristics derived from isotopic data (open ocean mixed-layer tropical/subtropical with symbionts, open ocean mixed-layer tropical/subtropical without symbionts, open ocean thermocline, open ocean sub-thermocline, high latitude)	Aze et al. [9]
Symbionts	Presence or absence of symbionts	Bé et al. [58]; Hemleben et al. [14]; Sen Gupta [59]; Feldman[60]; Kučera [17]; Coxall et al. [61]; Kasemann et al. [62]; Birch et al. [63]; Kučera et al. [64]; Ezard et al. [65]
Depth	Depth habitat which the species occupies (surface, surface-subsurface, subsurface, subsurface-deep, deep)	Bé et al. [58]; Hemleben et al. [14]; Sen Gupta [59]; Feldmann [60]; Kučera [17]; Coxall et al. [61]; Kasemann et al. [62]; Birch et al. [63]; Kučera et al. [64]; Ezard et al. [65]

#### Environmental data

Site-specific values of environmental variables were obtained from the sources listed in Table 2. The mixed-layer depth (i.e. the depth to which turbulence mixes the water effectively, and within which the temperature gradient is minimal) and the depth of the 10°C isotherm (Fig 2) were used to capture the vertical temperature structure. The mean annual Brunt-Väisälä frequency has been suggested as an alternative measure of vertical structure, which focusses more on the strength of the thermal gradient rather than the thickness of the mixed layer [66]. This metric was calculated for the top 300m of the water column from World Ocean Atlas Data [67, 68] using the Ocean Data View software [69]. However it was found to be strongly correlated with the sea-surface temperature (results not shown), so was not included in the analysis. Salinity levels in the ocean vary between 31 and 38 PSU [68]. The mean optimum salinity for planktonic foraminifera is estimated as 35.1 PSU [33], based on 20 macroperforate species. As the detrimental impact of salinity is expected to increase away from this optimum we use the absolute difference of the salinity at a site from this optimum to quantify salinity stress. Productivity was the only variable that was strongly skewed; we therefore log-transformed that variable to reduce the influence of high values.

**Table 2:** The environmental variables included in this analysis. Maps showing the variation in these variables are included in the supporting information (S1 File), along with information on the mean and range of the values across the sites. All variables are measured at 1 degree resolution; higher resolutions are available for some variables, but the data was too sparse to make it worthwhile including them.

Category	Variable	Description	Resolution	Source
Sea surface temperature	Mean SST	The annual mean sea-surface temperature	1 degree	World Ocean Atlas 09 [67]
Vertical temperature structure	Mixed-layer depth	The annual average depth to the base of the mixed layer, defined as a temperature change of 0.2°C from the surface temperature [70]	2 degree	IFREMER/LOS Mixed Layer Depth Climatology website (www.ifremer.fr/cerweb/deboyer/mld)
	10°C depth	The annual average depth to the 10°C isotherm (see Fig 2)	1 degree	Calculated from World Ocean Atlas 09 [67]
Seasonal assemblages	SD SST	The standard deviation in monthly sea-surface temperature	1 degree	World Ocean Atlas 09 [67]
	SD Salinity	The standard deviation in monthly salinity	1 degree	World Ocean Atlas 09 [68]
Productivity	Mean log Productivity	The annual mean of the logged net primary productivity [71]	1/6 degree, averaged to 1 degree	Ocean productivity (http://www.science.oregonstate.edu/ocean.productivity/index.php)
Stress	Mean Salinity	The annual mean salinity	1 degree	World Ocean Atlas 09 [68]
	Oxygen stress	The proportion of the year that a given location is at <2ml, measured at 100m depth	1 degree	World Ocean Atlas 09 [72]
Other	Ocean	The ocean in which the site is located	-	Kučera et al. [50]
	Dissolution	The bottom-water delta carbonate ion value, calculated using a spherical kriging model with the known relationship between ΔCO_3_^2-^ and water depth	1 degree	Archer [71]

Dissolution of carbonate occurs in water that is undersaturated in carbonate ions. We therefore use ΔCO_3_^2-^, a relative measure of carbonate saturation state at the sea floor [71], to quantify the risk of dissolution. If ΔCO_3_^2-^ > 0, the ocean floor is saturated with respect to carbonate and there is no risk of dissolution; we therefore set all positive values to zero before modelling. We excluded all sites with ΔCO_3_^2-^ < -10.9, the average value at the depth cut-offs used in previous studies to avoid dissolution bias (e.g. [12] excluded sites below 3500m in the Atlantic and below 4500m in the Pacific/Indian). Within the range 0 to -10.9, ΔCO_3_^2-^ therefore quantifies the undersaturation of carbonate. Alternative ΔCO_3_^2-^ values of -5 and -15 were tested, to determine the impact of this cut-off. The choice of value had relatively little impact (S1 Fig), although dissolution was a slightly more significant predictor of richness and lineage age when a more negative cut-off value was used.

The collinearity of variables was tested using the variance inflation factor (VIF). This metric regresses two variables against each other to identify collinearity. Where collinearity was detected (VIF > 5: [73]) the subordinate variable (that which explained less variance) was removed. Productivity and seasonality of production were highly collinear, meaning their importance cannot be separated. Sea-surface temperature was collinear with mean dissolved oxygen. For this reason, Tittensor et al. [12] instead measured oxygen stress in each of their 880km grid cells as the proportion of the cell with < 2ml per litre O_2_, a cut-off often taken to mark a significant impact on ocean life [74]. As our study’s spatial resolution is 1 degree, the same as the oxygen data, this exact measure would be binary; instead, we therefore measured oxygen stress as the proportion of the year spent below the 2ml per litre O_2_ threshold. No strong correlations were found between other variables for the full model.

In the few cases where environmental data were unavailable for a given grid cell (roughly 50 sites) the value for that variable was recorded as the mean of the values in the 8 surrounding cells. Sites where the surrounding cells were also missing data were excluded from the dataset. The dissolution measure is not available for the Mediterranean, which was therefore excluded from the analysis.

The environmental variables have a 1 degree resolution with the exception of mixed-layer depth, which is only available at 2 degree resolution. We therefore averaged the response data to the same 1 degree resolution as multiple points with the same explanatory variables would qualify as pseudoreplication, and increase the significance of the variables [75]. To test the impact of averaging, models were re-run 100 times, each time randomly sampling one site from each cell.

### Statistical methods

Bjørnstad and Falcks’s [76] bootstrapping method showed significant spatial autocorrelation in the residuals of ordinary least-squares models (results not shown), as expected in global analyses [77]. This autocorrelation was not adequately removed by generalised additive models with smooth terms for latitude and longitude [78], even with a high basis dimension for the smooth terms (results not shown), so we used simultaneous autoregressive models (SARs: [79, 80]). These are standard linear models with an added weights matrix to account for the spatial autocorrelation, which depends on the neighbourhood distances (distance over which the spatial autocorrelation acts) and the coding method (weighting given to neighbours). There are several types of SAR; Kissling and Carl [79] report that SAR_error_, which assumes the autoregressive process is in the error term, is the best method for ecological data such as ours.

We fitted a complete SAR_error_ model for all explanatory variables (see Table 2) and their two-way interactions for each of the response variables (SR, evenness, MEA and functional richness) globally. Dissolution was excluded from the interaction term, as the relationship between dissolution and diversity should be the same irrespective of the other environmental conditions. We selected the coding method (following [79]) and the optimal neighbourhood distance (between 500km and the distance where autocorrelation becomes non-significant, following [81]) using Akaike Information Criterion (AIC) comparisons for each model (see the results for full details of the parameters used).

To investigate the richness relationship in more detail, we further modelled each ocean separately. The full global model was too parameter-rich (seventy-five parameters) to fit robustly within each separate ocean. Stepwise model simplification of the full SR model, with a threshold p-value of 0.05 [82], was used to remove non-significant terms. Ocean terms were also removed. For each ocean, to ensure important interactions had not been missed, each interaction term was added back in turn with any significant terms retained in the model. Collinearity in the explanatory variables was tested for at each stage (S2 Fig shows log likelihood ratios for the full and simplified models). To compare the impact of more complete sampling, the Atlantic and Pacific were randomly subsampled, 100 times, to contain the same number of sites as the Indian Ocean.

As R^2^ is not calculated by SAR, Pseudo-R^2^, the squared Pearson correlation between the predicted and the observed values [79] was calculated using Nagelkerke’s [83] formula to measure model fit. The root mean squared error (RMSE), the average absolute departure of points from the fitted values, was also fitted.

Log-likelihood ratio tests were used to determine the contribution of each variable. Where subsampling had been used (i.e. for averaged sites in the main models, and to equalise sample sizes in the ocean models), variation in the estimates of the likelihood ratios was calculated for each variable (expressed on the subsequent plots as standard deviation).

Global diversity in the absence of dissolution (ΔCO_3_^2-^ = 0) was predicted using the final model coefficients applied to global layers of the environmental variables (see S1 File). Predictions were only made within the sampled ranges of the other environmental variables. Interpretation based on coefficients is challenging due to the interactions between the variables. Therefore, to investigate the shape of the relationships, each variable in turn was excluded from the models. The relative impact of its exclusion on model predictions was then investigated. Graphs showing how the diversity metric responded across the range of that variable were plotted.

Lastly, the MTE predictions for the relationship of SR and SST were tested by fitting a spatial autoregressive model with only SST (as a third-order polynomial) and ocean identity as explanatory variables, and with the same neighbourhood size and coding method as the global SR model. All analyses were done in the statistical environment R v. 3.0.3 [84]. Kriging was performed using the ‘gstat’ library [85]. SAR_error_ models were produced using the ‘spdep’ package [86].

## Results

**Fig 3:**
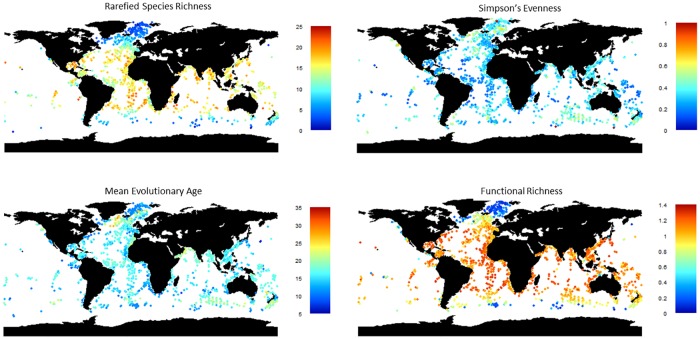
Diversity maps with the four different measures, showing the global spread of the data.

Fig 3 shows the observed diversity. SR peaks at 20° latitude, with a slight drop at the equator (as observed by [10]) and a steeper drop towards the poles. All three oceans are similar, although the limited data in the eastern Pacific makes it challenging to ascertain if there is an equatorial dip in SR in that region. Evenness is lower in tropical than in temperate or polar regions. MEA is lowest in polar regions and the Atlantic upwelling zones, and highest in sub-polar regions and the Indian upwelling zone. Functional richness has low polar and high tropical values, with a gradient much steeper than for SR and without an equatorial dip. With low SR, as in the high Atlantic sites, evolutionary age is heavily influenced by whether the dominant species is old (e.g. *Turborotalita quinqueloba*) or young (e.g. *Neogloboquadrina pachyderma*) (see Fig 1).

**Fig 4:**
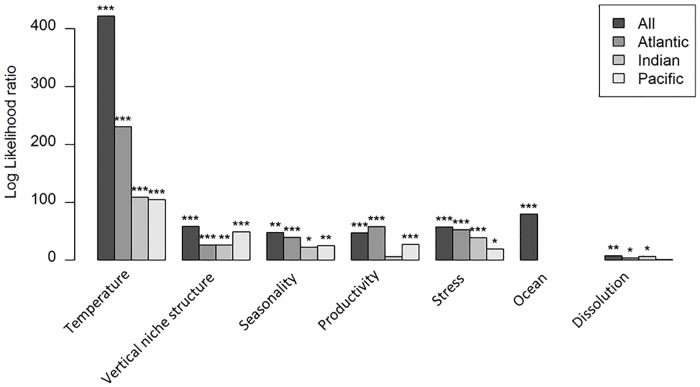
Log likelihood ratios for the species richness SAR_error_ model in each ocean. A comparison of the explanatory power of the groups of variables globally and in each ocean for the species richness model. Stars indicate the significance of excluding that variable group (*** < 0.001, 0.001 < ** < 0.01, 0.01 < * < 0.05, 0.05 <^**.**^ < 0.1). If relationships had the same functional form within each ocean, the total height of the bars for the three oceans would equal that of the global bar. The Atlantic model, with 670 data points, had a pseudo-R^2^ of 0.92, an RMSE of 1.59 and an AIC of 2510. The Indian model, with 155 data points, had a pseudo-R^2^ of 0.91, an RMSE of 1.38 and an AIC of 608. The Pacific model, with 235 data points, had a pseudo-R^2^ of 0.77 an RMSE of 1.82 and an AIC of 1024. All models used row-standardised weighting and a neighbourhood distance of 507km.

The full SAR_error_ model of SR had a pseudo-R^2^ of 0.89 and an RMSE of 1.74 species. The full model had an AIC of 4253 (for coefficients see S2 File), indicating a significantly better fit than the corresponding ordinary least squares model (AIC = 4313). This SAR_error_ model used row-standardised weighting and a neighbourhood distance of 507 km. For SR, temperature is the most important variable, followed by ocean, stress and vertical niche structure (Fig 4). The simplified model of SR (pseudo-R^2^ = 0.89, RMSE = 1.77, AIC = 4216), ascribes similar importance to the variable groups (Fig 4); differences probably result from the step-wise simplification. For these separate ocean models, explanatory power ranges from 92% (Atlantic) to 77% (Pacific). Temperature is highly significant for SR in all three oceans, especially the Atlantic (Fig 4). Productivity is the most different in effect between oceans: it is unimportant in the Indian but the second most important variable in the Atlantic. Caution is needed when interpreting these results as the number of data points widely varies between oceans. When sample size is standardised (S3 Fig), the variables become much less explanatory in the Atlantic, although the relative contributions of each variable remain similar for all three oceans.

**Fig 5:**
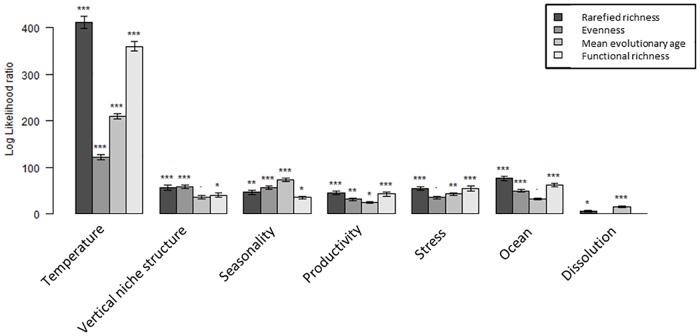
Log likelihood ratios for the SAR_error_ models of each diversity measure. The log likelihood ratios show the relative explanatory power of the groups of explanatory variables. This ratio is plotted for each variable group across the models of the four response variables. Error bars show 1sd and represent the variation associated with removing the replication within each 1 degree square.

The SAR_error_ models for the other variables were less explanatory (pseudo-R^2^ = 0.46 for evenness, 0.54 for average age, 0.85 for functional richness; RMSE = 0.096 for evenness, 2.35 million years for average age, 0.15 for functional richness). The relative importance of the variables in these models is shown in Fig 5. Functional richness has a model similar to that for SR although temperature is less important and dissolution is not significant; vertical niche structure is also less significant. Lineage age is mainly driven by temperature and seasonality, with no difference among oceans. Most variables are important for evenness, with temperature only slightly more important than the others, but stress and dissolution contribute little. The model residuals show no clear spatial patterns (S4 Fig).

S5 Fig shows the marginal effects of the different explanatory variables. Higher SR is associated with higher temperatures and moderate mixed-layer depths, particularly in the Indian Ocean. Interestingly the humped relationship of temperature with marginal SR found in the Atlantic is less clear in the other oceans. More seasonal waters have lower marginal SR. Temperature’s relationship with evenness is quite variable between the oceans. Higher values of salinity stress (i.e. further from the optimum) lead to more even assemblages in the Atlantic and Pacific, as do more productive and more seasonal environments. MEA is closely tied to SST, with the youngest ages at the lowest temperatures, followed by a peak of older communities at about 10°C and then a slight drop in evolutionary age. Age tends to increase with mixed-layer depth and increased variability. Functional richness shows much the same relationships as species richness, with the exception that increased seasonality is linked to higher functional richness but lower species richness. The relationship with salinity is also more clearly defined, with lower salinity stress being associated with the most functionally diverse communities.

**Fig 6:**
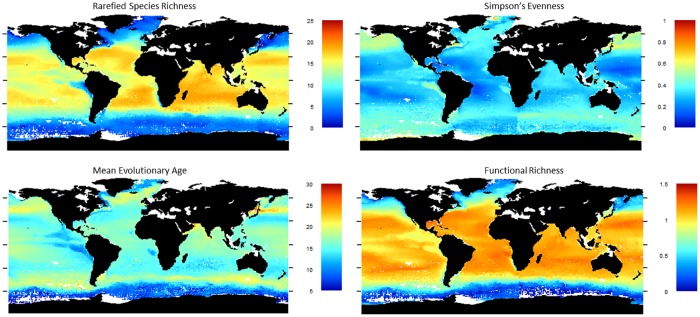
Predicted values of the diversity measures across the oceans.

Strong latitudinal patterns are seen in the fitted values of all the diversity measures (Fig 6). Longitudinal patterns also occur, such as in the eastern and the western Atlantic and either side of India. The more species rich assemblages in temperate regions are younger, while equatorial and sub-polar species-poor assemblages are typically older. Upwelling regions are modelled as having lower SR and functional diversity, as well as slightly younger assemblages than other sites at similar latitudes. The exception is the Indian upwelling region which has older species. The limitations of these predictions should be borne in mind. Pacific high-latitude predictions are supported by few data, as are the coastal regions, which tend to have different combinations of environmental variables from the open ocean. In models where the three oceans are considered separately, there are differences where the oceans meet; this difference is particularly noticeable for evenness.

**Fig 7:**
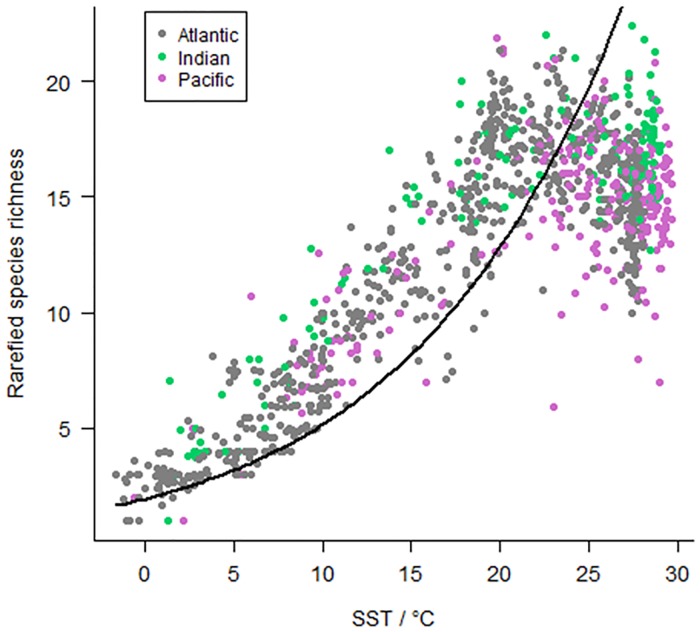
The relationship between rarefied species richness and SST (sea surface temperature) by ocean. The line shows the relationship predicted under the metabolic theory of ecology (MTE).

The relationship between SR and SST does not match the predictions of MTE: it varies significantly among oceans (p = 0.04) and does not have the predicted form in any ocean or overall (Fig 7).

## Discussion

Our results show many environmental factors independently explain significant geographic variation in coretop diversity in macroperforate planktonic foraminifera. Whereas previous analyses of Atlantic sites [10] found only sea surface temperature (SST) to be significant in multiple regression models, the structure of the Atlantic—with the high correlations among environmental variables—may have hampered a fuller exploration of the controls on diversity. When correlations between other variables and SST are weakened by including other oceans and multiple facets of diversity are investigated with a larger dataset, a more complex picture emerges. We consider the merits of the four sets of explanatory hypotheses outlined in the Introduction.

### Ecological limits

Ecological limits are argued to impose a limit on the diversity that can be sustained in a site. Such a limit could be imposed in a number of ways. If the oceanic niches are largely divided by depth, and each niche can only hold a limited number of species, more vertically-structured waters should have higher SR, functional richness and evenness. SR and functional richness do increase with vertical structure, but only up to a certain depth (S5 Fig), and evenness decreases if anything. So, either some other factor is influencing evenness, or the depth of the mixed layer is not a major driving force of the species richness patterns. However that does not completely rule out the importance of vertical temperature structure for diversity [10]. As mentioned above, our measures of vertical structure do not capture the strength of the stratification, as that is found to be strongly correlated with the SST. Given the significance of SST, these results imply stronger thermal gradients could increase diversity with niches being more strongly separated, but having a greater depth of the same conditions (i.e. a thicker mixed layer) does not increase the diversity.

Tittensor et al. [12] suggested that the off-equator peak in diversity could result from superposition of multiple assemblages due to seasonal fluctuations in ocean current boundaries. Alternatively seasonality could cause a reduction in specialist species. The highest SR sites (in the temperate regions) have relatively uneven assemblages while the equatorial sites have lower SR but also low evenness. Functional richness is equally high in both tropical and temperate regions, implying functional redundancy in warmer communities, particularly in the Indian and Pacific (Fig 6). If evenness reflects superposition of multiple assemblages [13], the relatively low evenness observed in the low to mid latitudes (Fig 6) imply seasonal turnover of diversity is not driving the diversity there. However the low richness observed in the more seasonally variable high latitudes is linked to more even assemblages, implying the communities that dominate these sites could vary through the year. Results from Žarić et al. [87], who studied seasonal variation in foraminiferal communities, provide some support for this theory, although species in mid-latitude communities mostly vary in abundance rather than disappearing entirely.

The other very seasonal sites are the highly productive coastal upwelling regions, which have low SR and evenness. This result suggests the superposition of seasonal communities does not dominate coastal upwelling assemblage composition (c.f. [32]); instead, such sites are likely to be dominated by generalists. This theory is supported by the high abundance of opportunistic species such as *Globigerina bulloides* in upwelling regions [88]. The Pacific and Atlantic upwelling assemblages are typically dominated by one of the two major groups within the clade, the omnivorous non-spinose globorotaliids. However the Indian upwelling contains a more equal mixture of spinose and non-spinose species (highlighted by the difference in MEA).

Neither oxygen stress nor salinity cause much change in SR across the range of values seen in our data, but there is a tendency towards more even communities at higher stress levels. There is a decrease in functional richness with both salinity and oxygen stress, suggesting certain functional groups are less able to cope with more extreme conditions. Planktonic foraminifera can tolerate the variations in salinity experienced in the oceans [89], but the more extreme conditions found in some ocean regions could slightly reduce survivorship or fecundity. Planktonic foraminifera may be particularly susceptible to slight reductions in fitness because, from what is known about their life history, Allee effects (a positive relationship between individual fitness and the density of conspecifics: [90]) could be very strong. It appears that species are semelparous, obligately sexual mass spawners, meaning that falls in local population size could greatly reduce reproductive success. Alternatively variations in salinity could be associated with other, unmodelled, environmental variables [24]. The interplay or these different conditions could make stress relationships harder to interpret.

Although SST is the most significant explanatory variable, it is measured more precisely than some of the others, which could elevate its relative importance over correlated variables in a multiple regression [91]. Thus, the inferred importance of the other explanatory variables may be underestimated.

### Evolutionary dynamics

The deep-time stability hypothesis receives support from the peaks in species richness within the subtropical gyres, perhaps the most stable environment on earth [92]. However, even though a global analysis of this clade’s macroevolution showed that environmental change has promoted extinction and high standing diversity has inhibited speciation [40], these gyre assemblages are not dominated by particularly old species. Environmental stability has not led to evolutionary stasis [93].

The youngest assemblages are those at very high latitudes dominated by *Neogloboquadrina pachyderma*; combined with their very low richness, these assemblages support the deep-time stability hypothesis. The oldest assemblages are in subpolar waters, which is where some of the older species reach their highest relative abundance (e.g. *Turborotalita quinqueloba*, *Globigerina bulloides*). This combination of moderately low richness, low functional diversity and old species does not fit either the deep-time stability or the out-of-the-tropics hypotheses. High mean species age implies phylogenetic overdispersion, perhaps suggesting a role for competitive interactions in structuring these subpolar assemblages [94], and perhaps also suggesting that the older species possess an incumbency advantage [95, 96] over newcomers. The four dominant morphospecies in these subpolar assemblages have speciated at much the same rate as other extant morphospecies (six speciation events in 98.7 Ma of lineage, giving a rate of 0.061/Ma, compared with an overall rate of 0.073/Ma for all Recent species), arguing against evolutionary source-sink dynamics [97] as the explanation for the region’s old assemblages (although this ignores any cryptic diversity).

Although the deep-time stability model receives only qualified support from our models, it may have been important for the gradual assembly of the gyre communities without leaving a strong signature on MEA, if species are still joining the community. The out-of-the-tropics model also receives little direct support from our models. However, fully resolving the importance of these hypotheses would require a spatiotemporal analysis of diversity dynamics, identifying where each species originated and mapping patterns in species persistence. Because many species in the clade’s history attained their global distribution very rapidly after formation, such resolution must await more precise dating [98].

The upwelling regions found along the eastern coasts and at the equator are highly seasonal, often highly productive, environments. Although these regions have persisted for a long time they tend to be species-poor: deep-time stability is not by itself sufficient to provide high richness. The Pacific and Atlantic upwelling regions are dominated by younger species, perhaps implying a rapid turnover with higher levels of extinction and speciation. However the Indian upwelling region contains relatively old species. This region differs from the Atlantic or Pacific as the upwelled water has a short residence time before it is dispersed, and upwelling ceases between the monsoonal periods [24]. The older community found in this environment could reflect an incumbency advantage of the species adapted to this more seasonal environment.

The metabolic theory of ecology (MTE) predicts the relationship between temperature and richness should be consistent in the three oceans; however our results show the oceans differ significantly in this relationship. There is also no evidence that speciation rates are higher at higher temperature for morphospecies (see above). The MTE predicts an exponential relationship between SST and richness, which is not observed (Fig 6), although the superposition of multiple communities in the sediment could mask that shape. Our results suggest that MTE does not underpin diversity patterns in this clade (though it may do so in bacteria: [47]).

### Alternative explanations and limitations

The statistical MDE predicts a peak in diversity at the centre of each ocean basin. Due to the different geographical ranges of these basins, there could be differences in results between oceans that are consistent with the MDE [25]. Although the pattern in richness is dominated by a latitudinal gradient, there is also marked longitudinal variation, which differs between ocean basins (Fig 6)–variation that purely spatial MDE models struggle to replicate [11, 26]. Alternatively an MDE has also been suggested to act on a temperature gradient to produce the observed mid-temperature diversity peak [26]. However although SR decreases clearly above 23°C in the Atlantic, this decrease is much less pronounced in the other oceans (S5 Fig), making it unlikely that diversity patterns result purely from an MDE acting on the temperature. Having removed sites based on the selected cut-off, dissolution explained little of the variation among the remaining sites.

Although an excellent study system in many ways, planktonic foraminifera present a number of challenges. The rapid attainment of wide distributions by new species makes pinpointing areas of origination difficult. Working with death assemblages conflates life span with instantaneous abundance, as well as inevitably superimposing assemblages from different depths and seasons. Correlations with productivity could be driven by either its mean or its seasonality, because the two are collinear in the marine realm. Nonetheless, planktonic foraminifera offer global sampling and a unique fossil record, giving a rare opportunity to consider the global macroecology of open-ocean plankton at the species level and to include evolutionary age among the facets of diversity considered. That same fossil record also provides a unique opportunity to test how well present-day macroecological correlates transfer to past environments—an obvious avenue for future research.

### Conclusions

We have tested a wide range of environmental factors that have been hypothesised to influence local coretop assemblage diversity in macroperforate planktonic foraminifera, using four different diversity measures (rarefied richness, Simpson’s evenness, functional richness and lineage age) to provide as complete an analysis as possible. Most of the ecological and evolutionary mechanisms that have been proposed to influence diversity received at least some support, but none fits all of the data. Sea-surface temperature is the strongest predictor of diversity; this metric is correlated with the strength of stratification in the water column. However the thickness of the mixed layer is not a strongly significant factor, suggesting the strength but not the depth of the water column structure contributes to diversity. Evenness suggests seasonal turnover could contribute to diversity at high latitudes, but there is little evidence for it in low to mid-latitudes. The seasonally variable upwelling regions in these latitudes show little turnover, instead relying on opportunistic species. Regions with less optimal environmental conditions tend to have lower functional diversity. The geologically stable gyres have high diversity, but the communities found there are relatively young implying there is little evolutionary stasis there. The oldest communities occur in the sub-polar regions, possibly suggesting competition, or an incumbency advantage, have contributed to their structure. The mechanisms that make the most precise predictions—MDE and MTE—are rejected as the sole explanations for the diversity patterns in this clade. Our results instead suggest that, as proposed by Gaston [99], observed diversity patterns are best explained by multiple factors and mechanisms acting in concert, and that the environmental correlations observed today have developed over geological timescales.

## Supporting Information

S1 Fig Log likelihood ratios for sites with different dissolution cut-offs. -10.9 was the cut-off used in this study.
(TIF)

S2 Fig Log likelihood ratios for the full SAR_error_ model, compared to the simplified model of rarefied species richness. The simplified version was produced to allow ocean level calculations of diversity.
(TIF)

S3 Fig Implications of reducing sampling to equal numbers of sites in the three oceans. The error bars are 1sd. For the full model, these error bars represent the variation associated with removing the replication within each 1 degree square. For the individual ocean models (Atlantic and Pacific), the error bars represent the variation associated with sampling the dataset to contain the same number of data points as the Indian. Consequently there are no error bars for the Indian.
(TIF)

S4 Fig Residuals from the full SAR_error_ models for the four response variables. (TIF)

S5 Fig Marginal effect of the explanatory variables on the diversity measure. The colours represent the three oceans: Black—Atlantic, Red—Indian, Blue—Pacific.
(TIF)

S1 File Maps of the explanatory variables used in the analysis. The environmental variables used in the analysis, and their ranges in the associated data.
(PDF)

S2 File Values of the coefficients and the likelihood ratios from the spatial autoregressive models. Coefficient summary for the full model of rarefied species richness. Coefficient summary for the full model of Simpson’s evenness. Coefficient summary for the full model of mean evolutionary age. Coefficient summary for the full model of Functional richness. Likelihood ratios and their significance for the different diversity models.
(PDF)

S1 Table Species list used in the analysis. Columns indicate the names in the MARGO dataset, the species used in this analysis and comments justifying the reasons. Coloured rows indicate definitions which were grouped.
(PDF)
