# Supplementary figures and images for: Environmental Predictors of Diversity in Recent Planktonic Foraminifera as Recorded in Marine Sediments
Source: PLoS One. 2016 Nov 16;11(11):e0165522. doi: 10.1371/journal.pone.0165522 (PMC5112986; doi:10.1371/journal.pone.0165522)

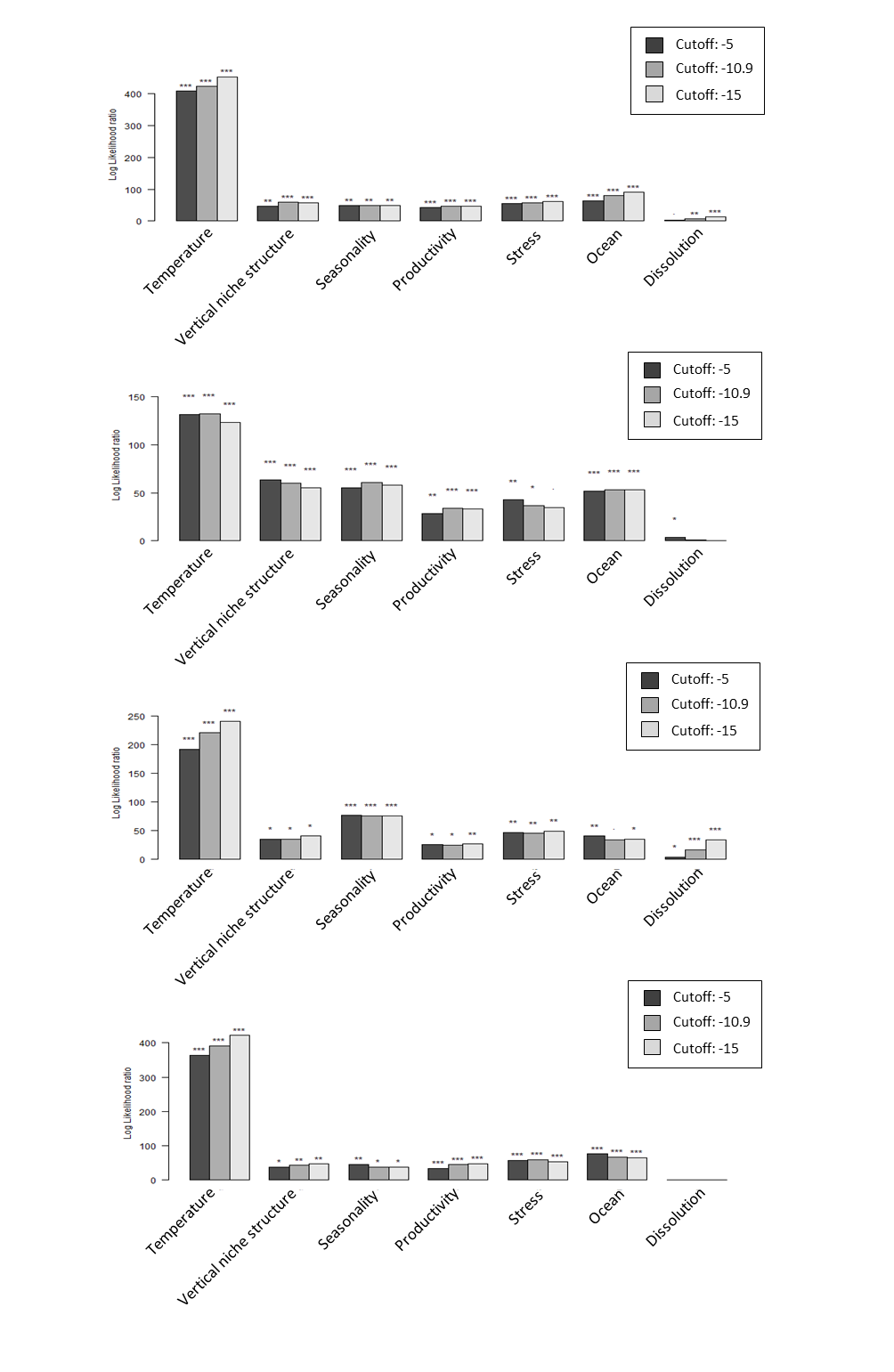

Supplement: S1 Fig — -10.9 was the cut-off used in this study. (TIF) [file pone.0165522.s001.tif]

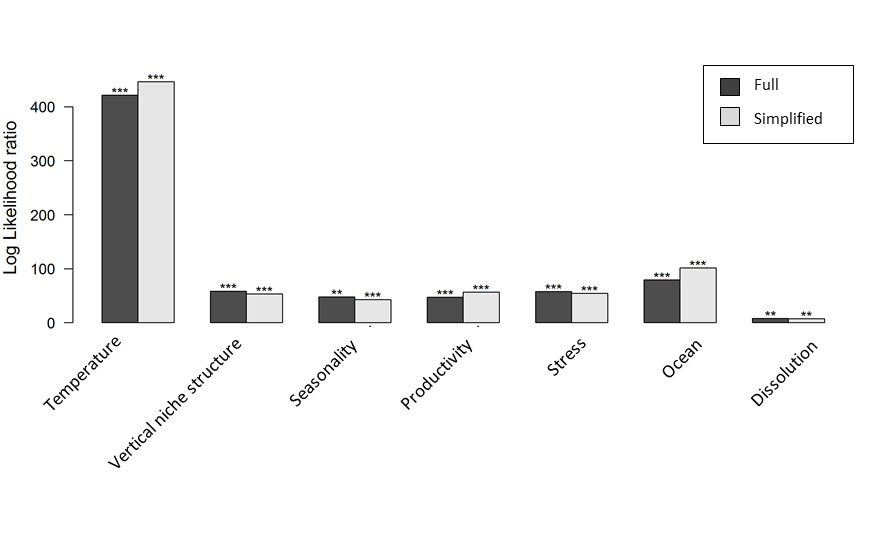

Supplement: S2 Fig — The simplified version was produced to allow ocean level calculations of diversity. (TIF) [file pone.0165522.s002.tif]

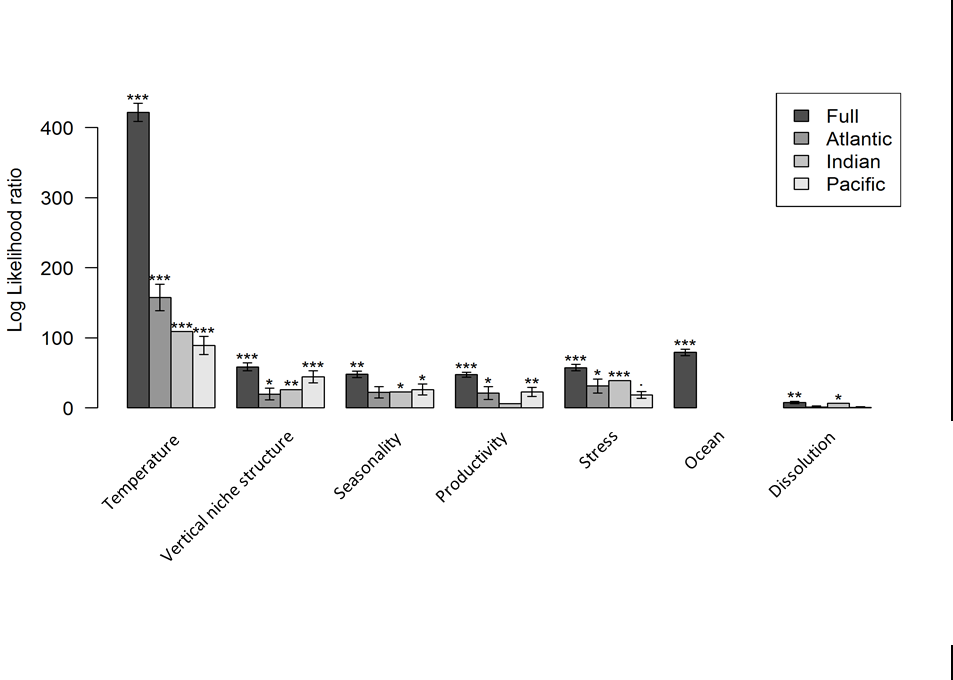

Supplement: S3 Fig — The error bars are 1sd. For the full model, these error bars represent the variation associated with removing the replication within each 1 degree square. For the individual ocean models (Atlantic and Pacific), the error bars represent the variation associated with sampling the dataset to contain the same number of data points as the Indian. Consequently there are no error bars for the Indian. (TIF) [file pone.0165522.s003.tif]

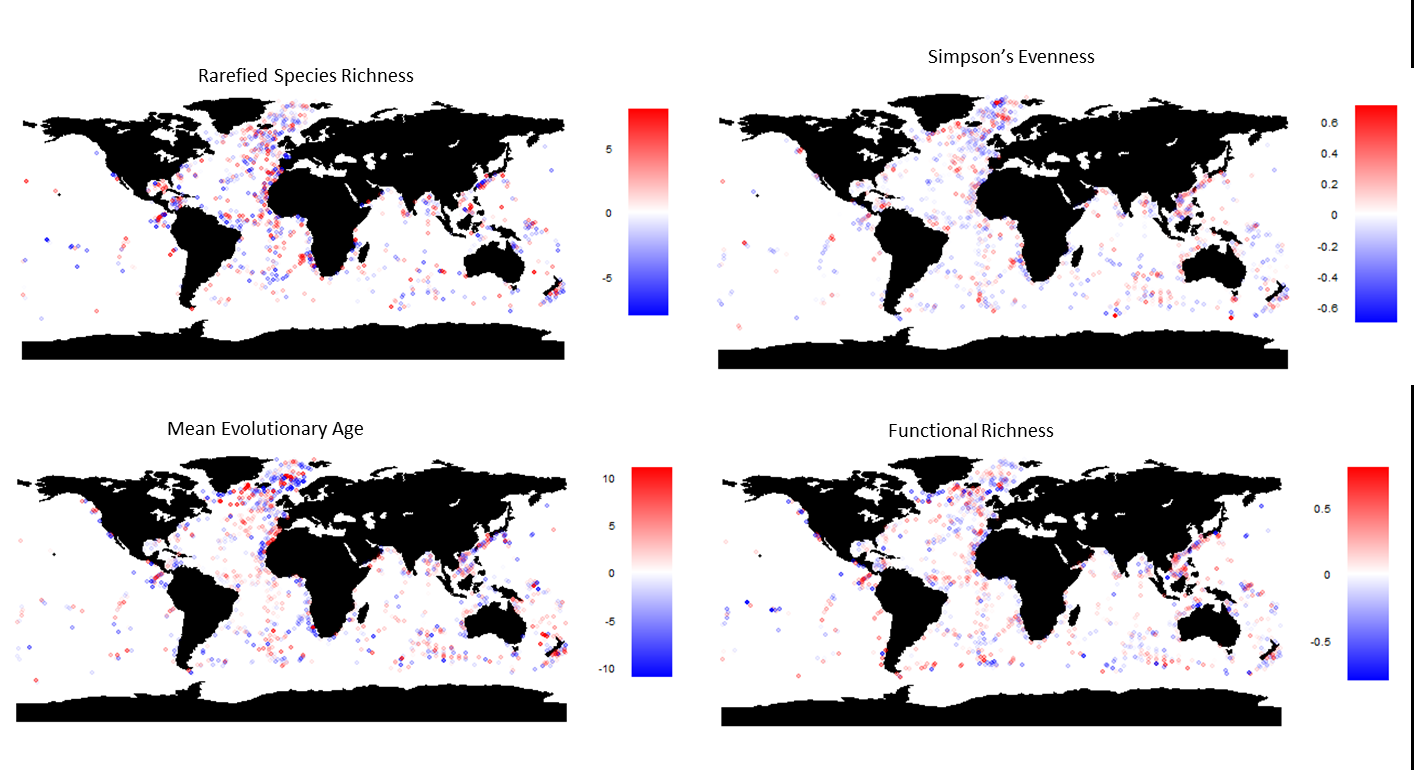

Supplement: S4 Fig — (TIF) [file pone.0165522.s004.tif]

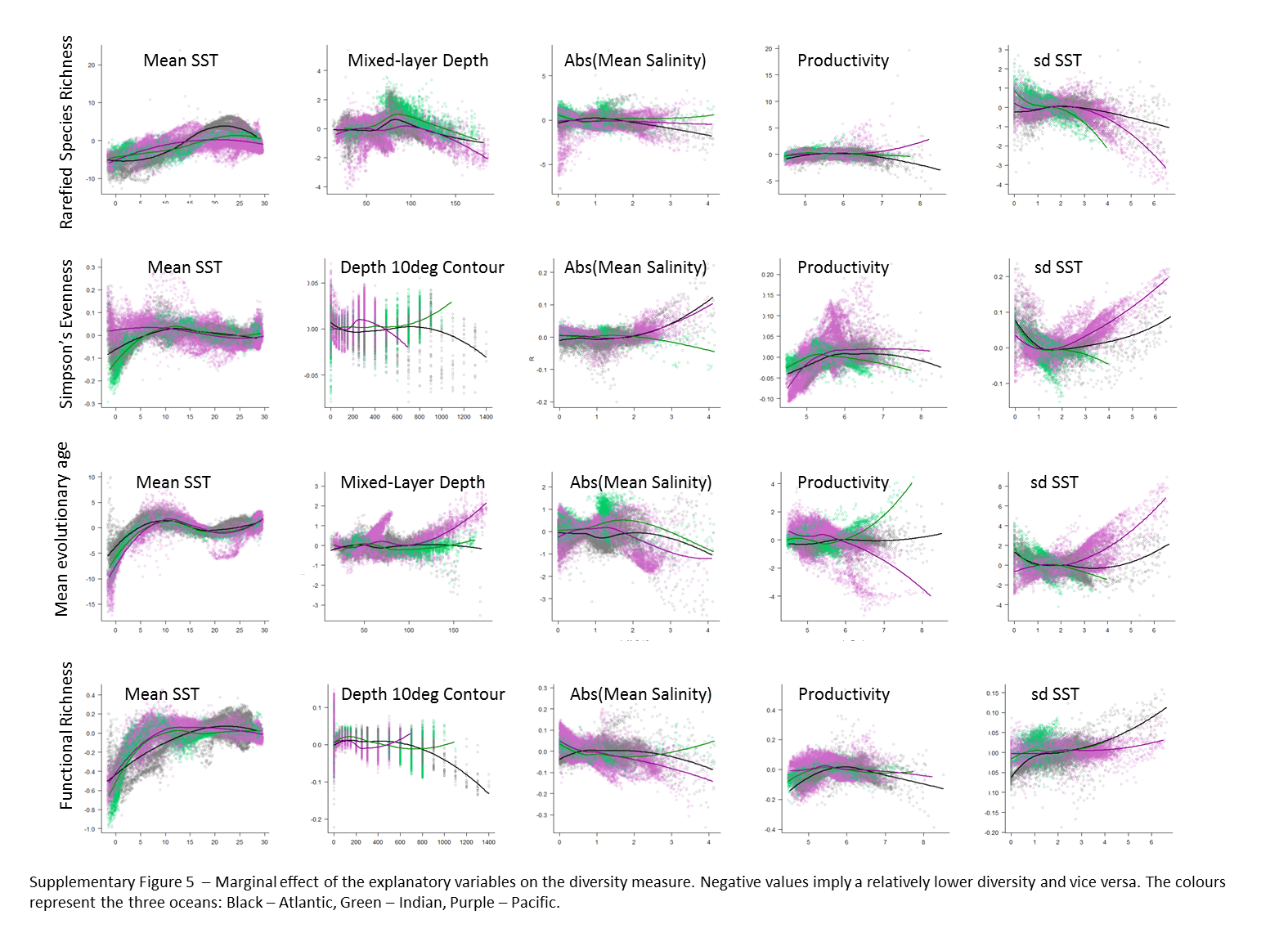

Supplement: S5 Fig — The colours represent the three oceans: Black—Atlantic, Red—Indian, Blue—Pacific. (TIF) [file pone.0165522.s005.tif]
