# Supplementary material for: Environmental Predictors of Diversity in Recent Planktonic Foraminifera as Recorded in Marine Sediments
Source: PLoS One. 2016 Nov 16;11(11):e0165522. doi: 10.1371/journal.pone.0165522 (PMC5112986; doi:10.1371/journal.pone.0165522)
Supplement: S1 File — The environmental variables used in the analysis, and their ranges in the associated data. (PDF) [file pone.0165522.s006.pdf]

Supporting Information

S1 Figure. Log likelihood ratios for sites with different dissolution cut-offs. -10.9 was the cut-off used in this study.

S2 Figure. Log likelihood ratios for the full SAR_{error} model, compared to the simplified model of rarefied species richness. The simplified version was produced to allow ocean level calculations of diversity.

S3 Figure. Implications of reducing sampling to equal numbers of sites in the three oceans. The error bars are 1sd. For the full model, these error bars represent the variation associated with removing the replication within each 1 degree square. For the individual ocean models (Atlantic and Pacific), the error bars represent the variation associated with sampling the dataset to contain the same number of data points as the Indian. Consequently there are no error bars for the Indian.

S4 Figure. Residuals from the full SAR_{error} models for the four response variables

S5 Figure. Marginal effect of the explanatory variables on the diversity measure. The colours represent the three oceans: Black – Atlantic, Red – Indian, Blue – Pacific.

S1 Table. Species list used in the analysis. Columns indicate the names in the MARGO dataset, the species used in this analysis and comments justifying the reasons. Coloured rows indicate definitions which were grouped.

S1 File. Maps of the explanatory variables used in the analysis. The environmental variables used in the analysis, and their ranges in the associated data.

S2 File. Values of the coefficients and the likelihood ratios from the spatial autoregressive models. Table 1: Coefficient summary for the full model of rarefied species richness. Table 2: Coefficient summary for the full model of Simpson's evenness. Table 3: Coefficient summary for the full model of mean evolutionary age. Table 4: Coefficient summary for the full model of Functional richness. Table 5: Likelihood ratios and their significance for the different diversity models.

S1 File. Maps of the explanatory variables used in the analysis. The environmental variables used in the analysis, and their ranges in the associated data.

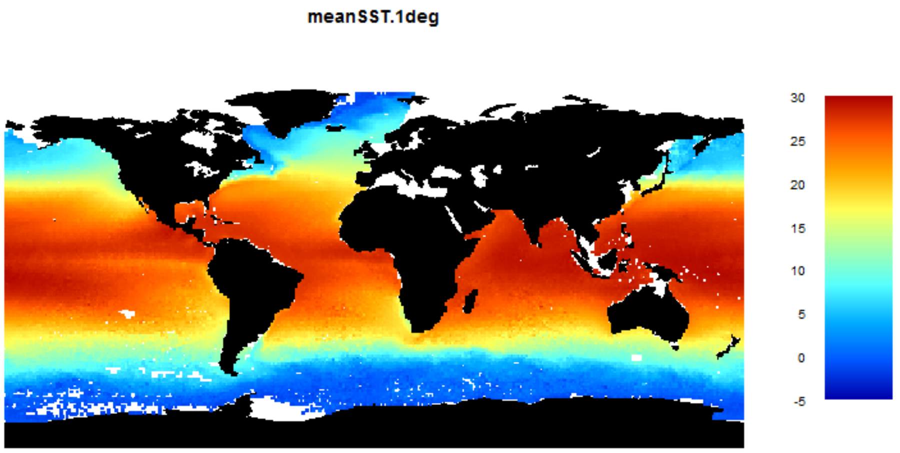

MARGO
Min: -1.90°C
Mean: 21.04°C
Max: 29.67°C

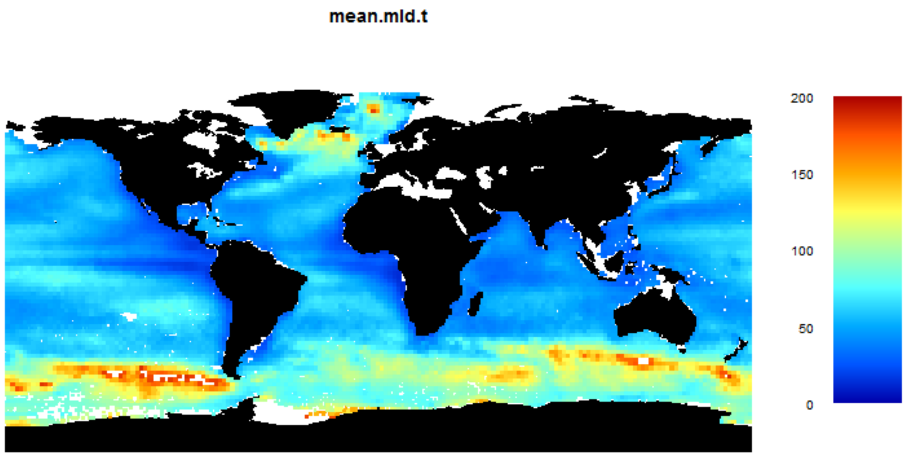

MARGO
Min: 12.42m
Mean: 56.85m
Max: 186.28m

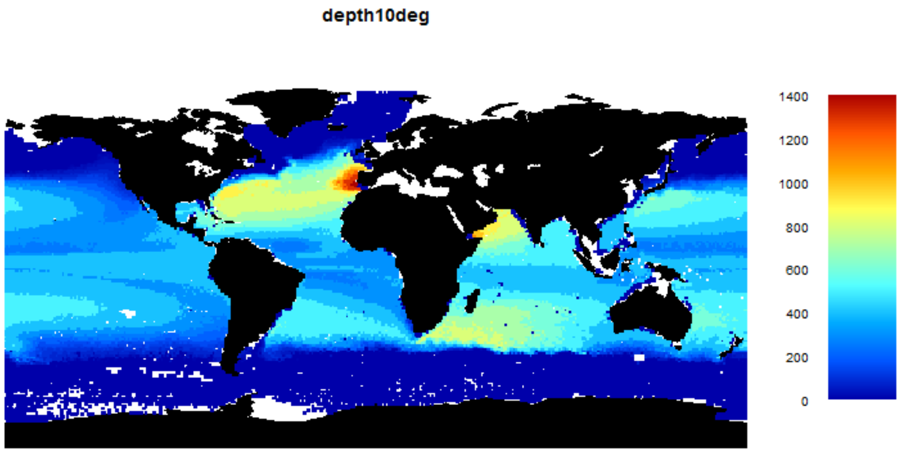

MARGO
Min: 0m
Mean: 361m
Max: 1400m

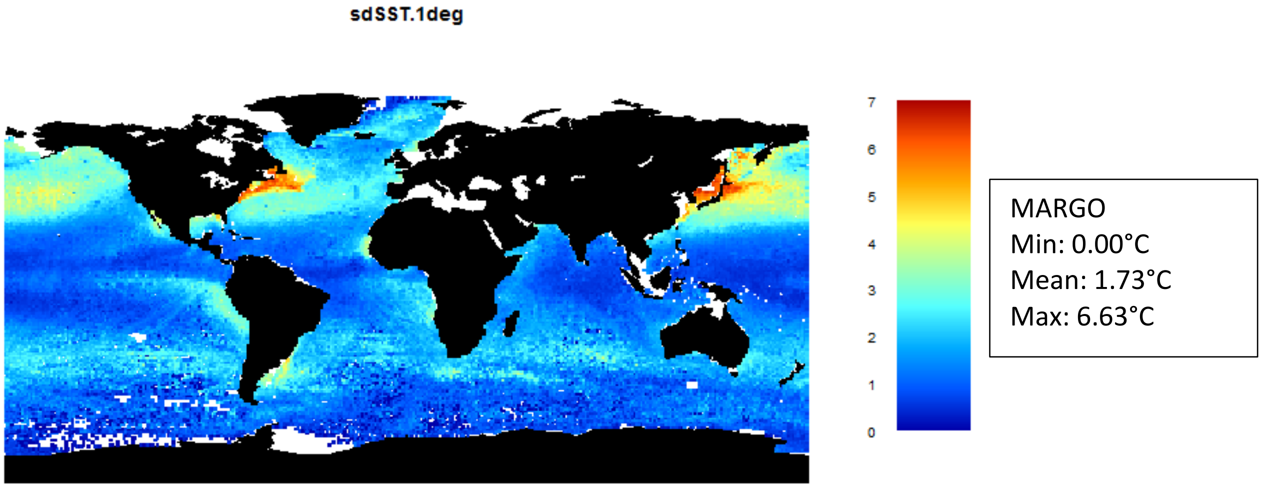
sdSST.1deg
7
6
5
4
3
2
1
0
MARGO
Min: 0.00°C
Mean: 1.73°C
Max: 6.63°C

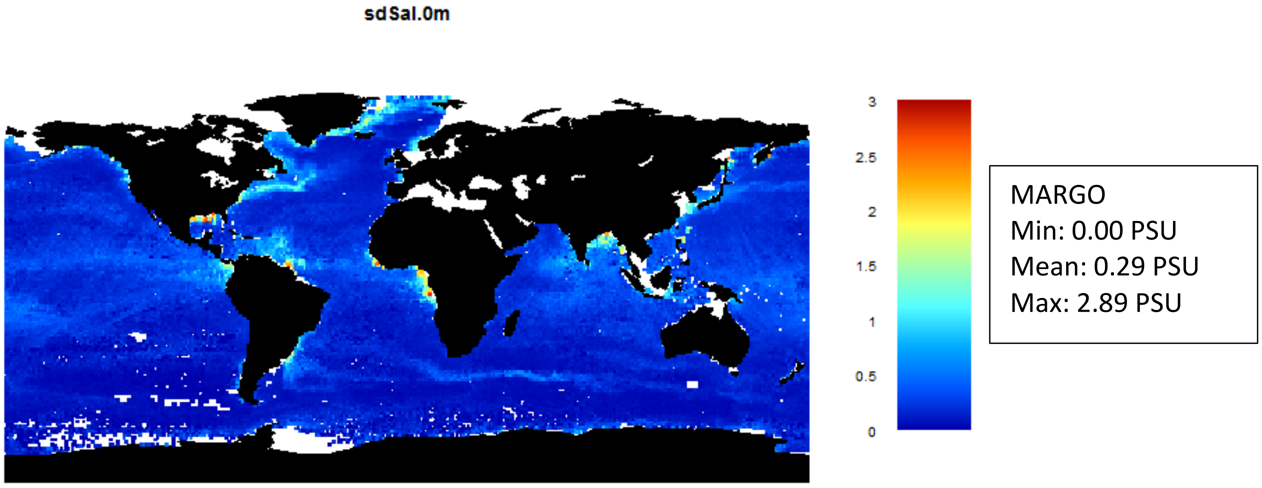
sdSal.0m
3
2.5
2
1.5
1
0.5
0
MARGO
Min: 0.00 PSU
Mean: 0.29 PSU
Max: 2.89 PSU

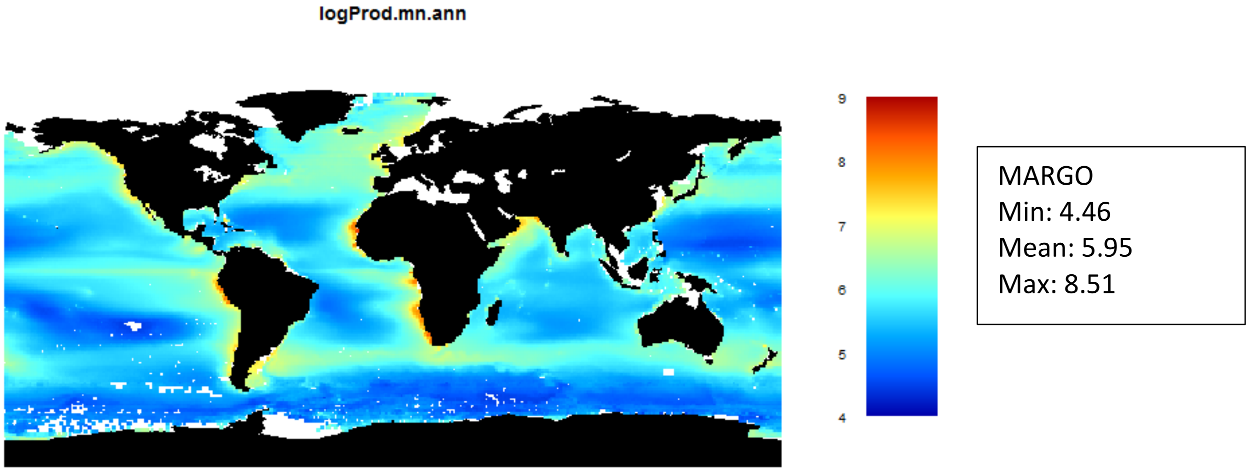
logProd.mn.ann
9
8
7
6
5
4
MARGO
Min: 4.46
Mean: 5.95
Max: 8.51

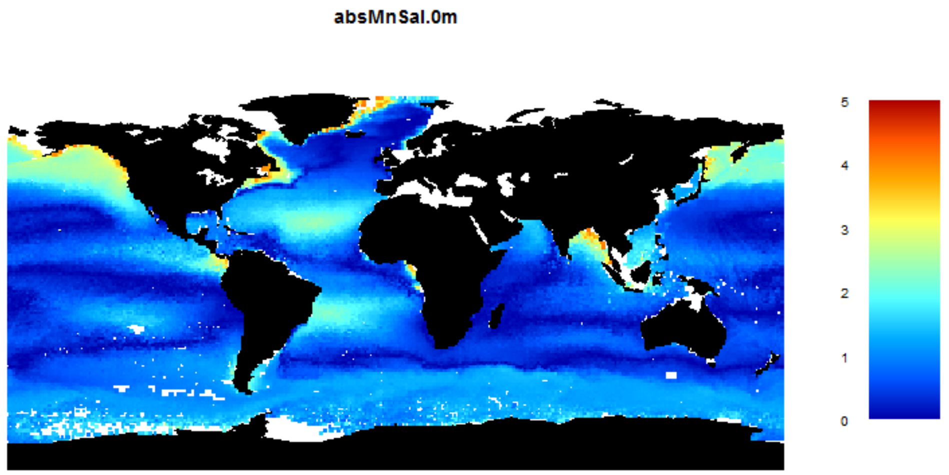

MARGO
Min: 0.00 PSU
Mean: 0.83 PSU
Max: 4.15 PSU

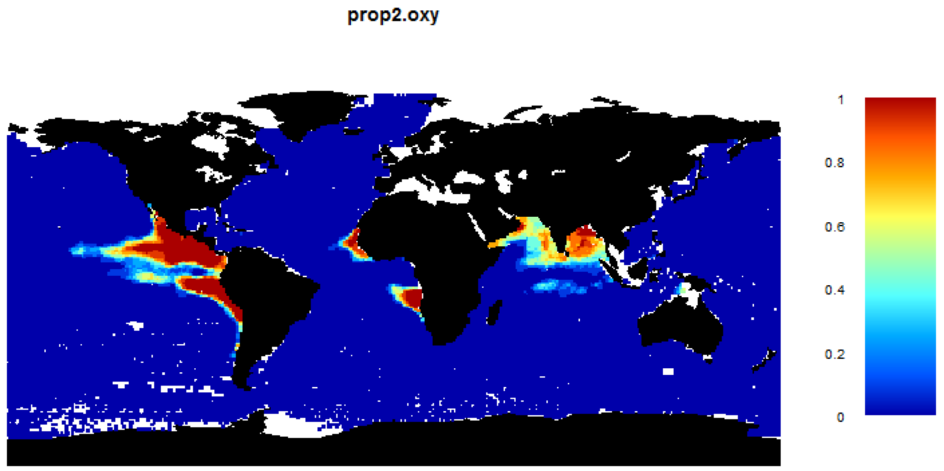

MARGO
Min: 0.00
Mean: 0.07
Max: 1.00
