# Supplementary material for: Environmental Predictors of Diversity in Recent Planktonic Foraminifera as Recorded in Marine Sediments
Source: PLoS One. 2016 Nov 16;11(11):e0165522. doi: 10.1371/journal.pone.0165522 (PMC5112986; doi:10.1371/journal.pone.0165522)
Supplement: S2 File — Coefficient summary for the full model of rarefied species richness. Coefficient summary for the full model of Simpson’s evenness. Coefficient summary for the full model of mean evolutionary age. Coefficient summary for the full model of Functional richness. Likelihood ratios and their significance for the different diversity models. (PDF) [file pone.0165522.s007.pdf]

Table 1 – Coefficient summary for the full model of rarefied species richness. Significance values: *** < 0.001, 0.001 < ** < 0.01, 0.01 < * < 0.05, 0.05 < ˙ < 0.1.

	**Estimate**	**Std. Error**	**z value**	**Pr(>|z|)**	**Significance**
(Intercept)	46.09	8.38	5.5	3.83E-08	***
poly(mnSST, 3)1	12.75	87.63	0.146	0.884	NA
poly(mnSST, 3)2	17.71	59.83	0.296	0.767	NA
poly(mnSST, 3)3	53.96	67.38	0.801	0.423	NA
sdSST	1.32	2.62	0.504	0.614	NA
I(MLD/10)	-3.8	0.925	-4.11	4.01E-05	***
I(depth10deg/100)	-0.168	1.01	-0.167	0.867	NA
mn.logProd	-5.17	1.3	-3.98	6.93E-05	***
absMnSal	-4.64	2.97	-1.56	0.119	NA
sdSal	-13.53	5.01	-2.7	0.00696	**
propOxy	54.76	29.16	1.88	0.0604	.
OceanIndian	-7.2	5.16	-1.4	0.162	NA
OceanPacific	-7.88	3.99	-1.97	0.0484	*
dCarbIon	0.0529	0.0186	2.85	0.00439	**
poly(mnSST, 3)1:sdSST	15.91	8.19	1.94	0.052	.
poly(mnSST, 3)2:sdSST	-1.6	5.29	-0.302	0.763	NA
poly(mnSST, 3)3:sdSST	-0.573	4.01	-0.143	0.886	NA
poly(mnSST, 3)1:I(MLD/10)	-2.94	3.22	-0.912	0.362	NA
poly(mnSST, 3)2:I(MLD/10)	-2.02	2.52	-0.8	0.424	NA
poly(mnSST, 3)3:I(MLD/10)	-0.631	2.13	-0.296	0.767	NA
poly(mnSST, 3)1:I(depth10deg/100)	1.92	15.97	0.12	0.905	NA
poly(mnSST, 3)2:I(depth10deg/100)	-2.35	10.36	-0.227	0.82	NA
poly(mnSST, 3)3:I(depth10deg/100)	-2.16	5.18	-0.417	0.676	NA
poly(mnSST, 3)1:mn.logProd	13.42	13.19	1.02	0.309	NA
poly(mnSST, 3)2:mn.logProd	-11.5	8.87	-1.3	0.195	NA
poly(mnSST, 3)3:mn.logProd	-16.37	9.74	-1.68	0.0928	.
poly(mnSST, 3)1:absMnSal	15.76	10.04	1.57	0.117	NA
poly(mnSST, 3)2:absMnSal	-19.71	8.26	-2.39	0.017	*
poly(mnSST, 3)3:absMnSal	-6.23	5.79	-1.08	0.282	NA
poly(mnSST, 3)1:sdSal	-9.99	20.83	-0.48	0.632	NA
poly(mnSST, 3)2:sdSal	71.52	19.67	3.64	0.000277	***
poly(mnSST, 3)3:sdSal	43.2	12.15	3.55	0.000379	***
poly(mnSST, 3)1:propOxy	-3049.57	1569.94	-1.94	0.0521	.
poly(mnSST, 3)2:propOxy	1903.81	1002.83	1.9	0.0576	.
poly(mnSST, 3)3:propOxy	-460.87	303.72	-1.52	0.129	NA
poly(mnSST, 3)1:OceanIndian	45.52	22.16	2.05	0.04	*
poly(mnSST, 3)2:OceanIndian	25.55	15.6	1.64	0.101	NA
poly(mnSST, 3)3:OceanIndian	0.759	14.94	0.0508	0.959	NA
poly(mnSST, 3)1:OceanPacific	19.02	16.62	1.14	0.252	NA
poly(mnSST, 3)2:OceanPacific	29.99	12.9	2.32	0.0201	*

poly(mnSST, 3)3:OceanPacific	17.9	10.32	1.73	0.0829	.
sdSST:I(MLD/10)	0.137	0.0694	1.98	0.0477	*
sdSST:I(depth10deg/100)	-0.046	0.0708	-0.651	0.515	NA
sdSST:mn.logProd	-0.314	0.403	-0.78	0.436	NA
sdSST:absMnSal	-0.0765	0.202	-0.38	0.704	NA
sdSST:sdSal	1.04	0.449	2.31	0.021	*
sdSST:propOxy	0.942	0.934	1.01	0.313	NA
sdSST:OceanIndian	-0.813	0.533	-1.53	0.127	NA
sdSST:OceanPacific	-0.297	0.362	-0.821	0.411	NA
I(MLD/10):I(depth10deg/100)	0.0224	0.0388	0.578	0.563	NA
I(MLD/10):mn.logProd	0.546	0.143	3.8	0.000142	***
I(MLD/10):absMnSal	0.227	0.126	1.81	0.0708	.
I(MLD/10):sdSal	-0.226	0.24	-0.945	0.345	NA
I(MLD/10):propOxy	-0.284	0.849	-0.335	0.738	NA
I(MLD/10):OceanIndian	0.419	0.193	2.17	0.0304	*
I(MLD/10):OceanPacific	0.262	0.148	1.77	0.0773	.
I(depth10deg/100):mn.logProd	0.00806	0.112	0.072	0.943	NA
I(depth10deg/100):absMnSal	-0.292	0.0864	-3.38	0.000738	***
I(depth10deg/100):sdSal	0.724	0.176	4.12	3.79E-05	***
I(depth10deg/100):propOxy	0.431	0.277	1.56	0.12	NA
I(depth10deg/100):OceanIndian	-0.153	0.163	-0.937	0.349	NA
I(depth10deg/100):OceanPacific	0.0302	0.139	0.217	0.828	NA
mn.logProd:absMnSal	0.839	0.419	2	0.0452	*
mn.logProd:sdSal	1.64	0.651	2.52	0.0118	*
mn.logProd:propOxy	0.284	1.08	0.263	0.793	NA
mn.logProd:OceanIndian	1.49	0.793	1.88	0.06	.
mn.logProd:OceanPacific	1.21	0.607	1.99	0.0461	*
absMnSal:sdSal	-0.297	0.367	-0.807	0.419	NA
absMnSal:propOxy	2.81	0.75	3.75	0.000179	***
absMnSal:OceanIndian	-1.35	0.668	-2.03	0.0428	*
absMnSal:OceanPacific	-0.698	0.502	-1.39	0.165	NA
sdSal:propOxy	-4.09	1.57	-2.6	0.00929	**
sdSal:OceanIndian	1.2	1.53	0.782	0.434	NA
sdSal:OceanPacific	0.302	1.13	0.268	0.789	NA
propOxy:OceanIndian	-3.94	1.96	-2.01	0.0447	*
propOxy:OceanPacific	-5.8	1.74	-3.32	0.000894	***

Table 2 - Coefficient summary for the full model of Simpson's evenness. Significance values: *** < 0.001, 0.001 < ** < 0.01, 0.01 < * < 0.05, 0.05 < ˙ < 0.1.

	Estimate	Std. Error	z value	Pr(>|z|)	Significance
(Intercept)	0.355	0.364	0.976	0.329	NA
poly(mnSST, 3)1	-5.63	4.44	-1.27	0.205	NA
poly(mnSST, 3)2	-10.19	2.93	-3.48	5.00E-04	***
poly(mnSST, 3)3	-2.45	3.07	-0.799	0.424	NA
sdSST	-0.0444	0.108	-0.412	0.68	NA
I(MLD/10)	-0.0376	0.0415	-0.905	0.366	NA
I(depth10deg/100)	-0.0733	0.042	-1.74	0.0811	.
mn.logProd	0.00956	0.0563	0.17	0.865	NA
absMnSal	-0.114	0.128	-0.893	0.372	NA
sdSal	0.0818	0.241	0.34	0.734	NA
propOxy	1.04	0.422	2.47	0.0136	*
OceanIndian	0.573	0.216	2.66	0.0079	**
OceanPacific	-0.0804	0.176	-0.456	0.649	NA
dCarbIon	-0.00082	0.000855	-0.954	0.34	NA
poly(mnSST, 3)1:sdSST	0.0399	0.417	0.0957	0.924	NA
poly(mnSST, 3)2:sdSST	-0.641	0.257	-2.49	0.0127	*
poly(mnSST, 3)3:sdSST	0.364	0.202	1.81	0.0708	.
poly(mnSST, 3)1:I(MLD/10)	-0.446	0.163	-2.73	0.00635	**
poly(mnSST, 3)2:I(MLD/10)	-0.166	0.132	-1.25	0.211	NA
poly(mnSST, 3)3:I(MLD/10)	-0.227	0.106	-2.14	0.032	*
poly(mnSST, 3)1:I(depth10deg/100)	1.38	0.784	1.76	0.078	.
poly(mnSST, 3)2:I(depth10deg/100)	-0.678	0.528	-1.28	0.199	NA
poly(mnSST, 3)3:I(depth10deg/100)	0.262	0.245	1.07	0.284	NA
poly(mnSST, 3)1:mn.logProd	1.01	0.689	1.46	0.144	NA
poly(mnSST, 3)2:mn.logProd	1.61	0.444	3.64	0.000275	***
poly(mnSST, 3)3:mn.logProd	0.633	0.452	1.4	0.162	NA
poly(mnSST, 3)1:absMnSal	-0.52	0.504	-1.03	0.302	NA
poly(mnSST, 3)2:absMnSal	0.498	0.384	1.3	0.194	NA
poly(mnSST, 3)3:absMnSal	0.209	0.287	0.729	0.466	NA
poly(mnSST, 3)1:sdSal	1.85	1.15	1.6	0.109	NA
poly(mnSST, 3)2:sdSal	0.626	1.01	0.62	0.536	NA
poly(mnSST, 3)3:sdSal	-1.33	0.625	-2.13	0.0329	*
poly(mnSST, 3)1:propOxy	-32.62	18.48	-1.77	0.0775	.
poly(mnSST, 3)2:propOxy	20.13	14.25	1.41	0.158	NA
poly(mnSST, 3)3:propOxy	-7.33	5.36	-1.37	0.171	NA
poly(mnSST, 3)1:OceanIndian	-0.465	0.973	-0.478	0.633	NA
poly(mnSST, 3)2:OceanIndian	-0.18	0.696	-0.259	0.796	NA
poly(mnSST, 3)3:OceanIndian	0.885	0.617	1.43	0.152	NA
poly(mnSST, 3)1:OceanPacific	1.04	0.793	1.31	0.19	NA
poly(mnSST, 3)2:OceanPacific	2.01	0.607	3.3	0.000965	***

poly(mnSST, 3)3:OceanPacific	0.316	0.471	0.672	0.502	NA
sdSST:I(MLD/10)	0.00089	0.00318	0.28	0.779	NA
sdSST:I(depth10deg/100)	0.00125	0.00299	0.419	0.676	NA
sdSST:mn.logProd	0.00403	0.0163	0.247	0.805	NA
sdSST:absMnSal	0.0167	0.0093	1.8	0.0725	.
sdSST:sdSal	-0.0144	0.0203	-0.71	0.477	NA
sdSST:propOxy	0.0784	0.0408	1.92	0.0549	.
sdSST:OceanIndian	-0.0331	0.0209	-1.58	0.113	NA
sdSST:OceanPacific	0.0239	0.0161	1.48	0.138	NA
I(MLD/10):I(depth10deg/100)	0.00232	0.00165	1.41	0.158	NA
I(MLD/10):mn.logProd	0.00366	0.00651	0.563	0.573	NA
I(MLD/10):absMnSal	0.00113	0.00553	0.204	0.838	NA
I(MLD/10):sdSal	0.0253	0.0117	2.17	0.0302	*
I(MLD/10):propOxy	0.0255	0.0369	0.69	0.49	NA
I(MLD/10):OceanIndian	-0.00585	0.00793	-0.737	0.461	NA
I(MLD/10):OceanPacific	0.00808	0.00699	1.16	0.248	NA
I(depth10deg/100):mn.logProd	0.0056	0.00479	1.17	0.242	NA
I(depth10deg/100):absMnSal	0.00761	0.00383	1.99	0.0467	*
I(depth10deg/100):sdSal	-0.00217	0.00829	-0.262	0.793	NA
I(depth10deg/100):propOxy	-0.00967	0.0101	-0.959	0.338	NA
I(depth10deg/100):OceanIndian	0.00215	0.00642	0.335	0.738	NA
I(depth10deg/100):OceanPacific	-0.00582	0.00543	-1.07	0.284	NA
mn.logProd:absMnSal	0.0108	0.0181	0.597	0.551	NA
mn.logProd:sdSal	-0.0322	0.0312	-1.03	0.303	NA
mn.logProd:propOxy	-0.0912	0.0449	-2.03	0.042	*
mn.logProd:OceanIndian	-0.0747	0.0331	-2.26	0.024	*
mn.logProd:OceanPacific	0.0138	0.0266	0.518	0.605	NA
absMnSal:sdSal	0.0178	0.0169	1.05	0.292	NA
absMnSal:propOxy	-0.0211	0.0332	-0.634	0.526	NA
absMnSal:OceanIndian	-0.0197	0.0286	-0.687	0.492	NA
absMnSal:OceanPacific	-0.00189	0.0217	-0.0869	0.931	NA
sdSal:propOxy	-0.028	0.0703	-0.398	0.69	NA
sdSal:OceanIndian	0.0141	0.0658	0.214	0.83	NA
sdSal:OceanPacific	-0.08	0.0482	-1.66	0.0974	.
propOxy:OceanIndian	-0.0086	0.0866	-0.0994	0.921	NA
propOxy:OceanPacific	0.00137	0.0736	0.0187	0.985	NA

Table 3 - Coefficient summary for the full model of mean evolutionary age. Significance values: *** < 0.001, 0.001 < ** < 0.01, 0.01 < * < 0.05, 0.05 < . < 0.1.

	Estimate	Std. Error	z value	Pr(>|z|)	Significance
(Intercept)	22.41	9.21	2.43	0.015	*
poly(mnSST, 3)1	-152.47	113.27	-1.35	0.178	NA
poly(mnSST, 3)2	-124.75	73.5	-1.7	0.0896	.
poly(mnSST, 3)3	24.05	77.42	0.311	0.756	NA
sdSST	-4.47	2.73	-1.64	0.101	NA
I(MLD/10)	-0.253	1.07	-0.237	0.813	NA
I(depth10deg/100)	0.812	1.05	0.776	0.438	NA
mn.logProd	-1.2	1.43	-0.843	0.399	NA
absMnSal	-3.41	3.31	-1.03	0.303	NA
sdSal	4.67	5.95	0.786	0.432	NA
propOxy	-6.28	11.17	-0.562	0.574	NA
OceanIndian	-2.75	5.93	-0.464	0.642	NA
OceanPacific	5.58	4.67	1.2	0.232	NA
dCarbIon	0.0856	0.0205	4.18	2.95E-05	***
poly(mnSST, 3)1:sdSST	-4.23	10.62	-0.399	0.69	NA
poly(mnSST, 3)2:sdSST	2.99	6.47	0.462	0.644	NA
poly(mnSST, 3)3:sdSST	27.01	5.17	5.22	1.76E-07	***
poly(mnSST, 3)1:I(MLD/10)	3.18	4.23	0.752	0.452	NA
poly(mnSST, 3)2:I(MLD/10)	4.93	3.37	1.46	0.144	NA
poly(mnSST, 3)3:I(MLD/10)	4.42	2.7	1.63	0.102	NA
poly(mnSST, 3)1:I(depth10deg/100)	-0.0124	19.24	-0.00065	0.999	NA
poly(mnSST, 3)2:I(depth10deg/100)	1.84	13.09	0.141	0.888	NA
poly(mnSST, 3)3:I(depth10deg/100)	-4.85	6.18	-0.786	0.432	NA
poly(mnSST, 3)1:mn.logProd	28.04	17.53	1.6	0.11	NA
poly(mnSST, 3)2:mn.logProd	15.4	11.08	1.39	0.164	NA
poly(mnSST, 3)3:mn.logProd	-0.206	11.41	-0.0181	0.986	NA
poly(mnSST, 3)1:absMnSal	5.89	12.98	0.453	0.65	NA
poly(mnSST, 3)2:absMnSal	-13.63	10.14	-1.34	0.179	NA
poly(mnSST, 3)3:absMnSal	-21.44	7.34	-2.92	0.00348	**
poly(mnSST, 3)1:sdSal	11.84	28.83	0.411	0.681	NA
poly(mnSST, 3)2:sdSal	4.59	25.33	0.181	0.856	NA
poly(mnSST, 3)3:sdSal	-62.92	16.28	-3.86	0.000111	***
poly(mnSST, 3)1:propOxy	-611.01	449.43	-1.36	0.174	NA
poly(mnSST, 3)2:propOxy	399.14	348.31	1.15	0.252	NA
poly(mnSST, 3)3:propOxy	-64.69	132.34	-0.489	0.625	NA
poly(mnSST, 3)1:OceanIndian	-12.03	26.39	-0.456	0.649	NA
poly(mnSST, 3)2:OceanIndian	-6.45	19.79	-0.326	0.744	NA
poly(mnSST, 3)3:OceanIndian	18.8	17.34	1.08	0.278	NA
poly(mnSST, 3)1:OceanPacific	21.88	22.2	0.986	0.324	NA
poly(mnSST, 3)2:OceanPacific	-7.65	17.66	-0.433	0.665	NA
poly(mnSST, 3)3:OceanPacific	6.65	13.53	0.492	0.623	NA

sdSST:I(MLD/10)	0.0787	0.0805	0.977	0.329	NA
sdSST:I(depth10deg/100)	-0.0538	0.0734	-0.732	0.464	NA
sdSST:mn.logProd	0.746	0.414	1.8	0.0716	.
sdSST:absMnSal	-0.15	0.235	-0.64	0.522	NA
sdSST:sdSal	-0.593	0.518	-1.14	0.253	NA
sdSST:propOxy	0.868	1.04	0.836	0.403	NA
sdSST:OceanIndian	-0.605	0.515	-1.17	0.24	NA
sdSST:OceanPacific	0.881	0.432	2.04	0.0414	*
I(MLD/10):I(depth10deg/100)	-0.0294	0.0414	-0.711	0.477	NA
I(MLD/10):mn.logProd	0.0514	0.167	0.307	0.759	NA
I(MLD/10):absMnSal	-0.0661	0.143	-0.461	0.645	NA
I(MLD/10):sdSal	-0.0311	0.288	-0.108	0.914	NA
I(MLD/10):propOxy	-0.616	0.965	-0.638	0.523	NA
I(MLD/10):OceanIndian	0.0629	0.206	0.305	0.761	NA
I(MLD/10):OceanPacific	0.163	0.179	0.912	0.362	NA
I(depth10deg/100):mn.logProd	-0.0938	0.119	-0.786	0.432	NA
I(depth10deg/100):absMnSal	0.042	0.0959	0.438	0.662	NA
I(depth10deg/100):sdSal	0.0673	0.201	0.335	0.737	NA
I(depth10deg/100):propOxy	0.123	0.258	0.478	0.633	NA
I(depth10deg/100):OceanIndian	-0.15	0.167	-0.896	0.37	NA
I(depth10deg/100):OceanPacific	-0.268	0.138	-1.94	0.052	.
mn.logProd:absMnSal	0.644	0.466	1.38	0.167	NA
mn.logProd:sdSal	-0.786	0.788	-0.997	0.319	NA
mn.logProd:propOxy	2.15	1.21	1.77	0.0762	.
mn.logProd:OceanIndian	0.732	0.922	0.794	0.427	NA
mn.logProd:OceanPacific	-1.35	0.716	-1.89	0.0586	.
absMnSal:sdSal	0.19	0.42	0.452	0.651	NA
absMnSal:propOxy	-0.108	0.871	-0.124	0.901	NA
absMnSal:OceanIndian	0.0118	0.764	0.0154	0.988	NA
absMnSal:OceanPacific	0.221	0.589	0.374	0.708	NA
sdSal:propOxy	1.5	1.78	0.846	0.397	NA
sdSal:OceanIndian	-0.597	1.64	-0.364	0.716	NA
sdSal:OceanPacific	1.1	1.21	0.91	0.363	NA
propOxy:OceanIndian	4.42	2.3	1.92	0.0553	.
propOxy:OceanPacific	0.526	2	0.263	0.793	NA

Table 4 - Coefficient summary for the full model of Functional richness. Significance values: *** < 0.001, 0.001 < ** < 0.01, 0.01 < * < 0.05, 0.05 < ˙ < 0.1.

	**Estimate**	**Std. Error**	**z value**	**Pr(>|z|)**	**Significance**
(Intercept)	2.13	0.522	4.07	4.64E-05	***
poly(mnSST, 3)1	1.45	6.28	0.231	0.817	NA
poly(mnSST, 3)2	-5.16	4.15	-1.24	0.214	NA
poly(mnSST, 3)3	8.71	4.33	2.01	0.0442	*
sdSST	0.212	0.155	1.36	0.173	NA
I(MLD/10)	-0.11	0.06	-1.83	0.0674	.
I(depth10deg/100)	-0.0326	0.0592	-0.55	0.582	NA
mn.logProd	-0.189	0.0805	-2.34	0.0191	*
absMnSal	-0.55	0.188	-2.93	0.00338	**
sdSal	-0.0466	0.352	-0.132	0.895	NA
propOxy	0.0666	0.576	0.116	0.908	NA
OceanIndian	0.292	0.314	0.93	0.352	NA
OceanPacific	0.18	0.254	0.709	0.478	NA
dCarbIon	0.000153	0.00122	0.125	0.9	NA
poly(mnSST, 3)1:sdSST	-0.811	0.591	-1.37	0.17	NA
poly(mnSST, 3)2:sdSST	-0.265	0.364	-0.728	0.466	NA
poly(mnSST, 3)3:sdSST	0.0503	0.278	0.181	0.857	NA
poly(mnSST, 3)1:I(MLD/10)	-0.32	0.234	-1.37	0.171	NA
poly(mnSST, 3)2:I(MLD/10)	-0.299	0.191	-1.56	0.118	NA
poly(mnSST, 3)3:I(MLD/10)	-0.284	0.147	-1.93	0.054	.
poly(mnSST, 3)1:I(depth10deg/100)	-1.32	1.03	-1.28	0.2	NA
poly(mnSST, 3)2:I(depth10deg/100)	1.46	0.698	2.1	0.0358	*
poly(mnSST, 3)3:I(depth10deg/100)	-0.436	0.332	-1.31	0.189	NA
poly(mnSST, 3)1:mn.logProd	1.14	0.967	1.18	0.237	NA
poly(mnSST, 3)2:mn.logProd	0.0718	0.618	0.116	0.907	NA
poly(mnSST, 3)3:mn.logProd	-1.42	0.635	-2.24	0.0253	*
poly(mnSST, 3)1:absMnSal	1.62	0.756	2.15	0.0316	*
poly(mnSST, 3)2:absMnSal	0.659	0.553	1.19	0.233	NA
poly(mnSST, 3)3:absMnSal	0.374	0.409	0.913	0.361	NA
poly(mnSST, 3)1:sdSal	1.31	1.7	0.77	0.441	NA
poly(mnSST, 3)2:sdSal	3.69	1.45	2.55	0.0108	*
poly(mnSST, 3)3:sdSal	-1.05	0.868	-1.21	0.226	NA
poly(mnSST, 3)1:propOxy	4.06	24.13	0.168	0.866	NA
poly(mnSST, 3)2:propOxy	-8.99	18.98	-0.474	0.636	NA
poly(mnSST, 3)3:propOxy	5.55	7.27	0.764	0.445	NA
poly(mnSST, 3)1:OceanIndian	0.274	1.36	0.2	0.841	NA
poly(mnSST, 3)2:OceanIndian	-1.48	0.98	-1.51	0.13	NA
poly(mnSST, 3)3:OceanIndian	0.871	0.869	1	0.316	NA
poly(mnSST, 3)1:OceanPacific	-1.36	1.14	-1.19	0.234	NA
poly(mnSST, 3)2:OceanPacific	0.56	0.908	0.617	0.537	NA

poly(mnSST, 3)3:OceanPacific	0.451	0.716	0.63	0.529	NA
sdSST:I(MLD/10)	-0.00286	0.0047	-0.607	0.544	NA
sdSST:I(depth10deg/100)	0.00059	0.0043	0.137	0.891	NA
sdSST:mn.logProd	-0.0293	0.0235	-1.25	0.211	NA
sdSST:absMnSal	-0.0124	0.0138	-0.904	0.366	NA
sdSST:sdSal	0.0539	0.0296	1.82	0.0684	.
sdSST:propOxy	0.024	0.0581	0.413	0.68	NA
sdSST:OceanIndian	-0.0194	0.0299	-0.648	0.517	NA
sdSST:OceanPacific	-0.0302	0.0232	-1.3	0.192	NA
I(MLD/10):I(depth10deg/100)	-0.00044	0.00235	-0.188	0.851	NA
I(MLD/10):mn.logProd	0.016	0.00938	1.71	0.0878	.
I(MLD/10):absMnSal	0.0228	0.00852	2.68	0.00737	**
I(MLD/10):sdSal	-0.023	0.0177	-1.3	0.194	NA
I(MLD/10):propOxy	-0.0248	0.0525	-0.472	0.637	NA
I(MLD/10):OceanIndian	0.00318	0.0114	0.279	0.78	NA
I(MLD/10):OceanPacific	0.00536	0.01	0.536	0.592	NA
I(depth10deg/100):mn.logProd	0.0102	0.00685	1.49	0.136	NA
I(depth10deg/100):absMnSal	-0.00625	0.00548	-1.14	0.254	NA
I(depth10deg/100):sdSal	0.0157	0.0119	1.32	0.186	NA
I(depth10deg/100):propOxy	-0.0094	0.0144	-0.652	0.515	NA
I(depth10deg/100):OceanIndian	-0.00897	0.00926	-0.968	0.333	NA
I(depth10deg/100):OceanPacific	0.00753	0.00778	0.967	0.333	NA
mn.logProd:absMnSal	0.089	0.026	3.42	0.000623	***
mn.logProd:sdSal	-0.0131	0.0448	-0.294	0.769	NA
mn.logProd:propOxy	-0.00527	0.0639	-0.0824	0.934	NA
mn.logProd:OceanIndian	-0.0361	0.048	-0.752	0.452	NA
mn.logProd:OceanPacific	-0.03	0.0385	-0.778	0.437	NA
absMnSal:sdSal	-0.0366	0.0248	-1.48	0.139	NA
absMnSal:propOxy	0.0803	0.0475	1.69	0.0912	.
absMnSal:OceanIndian	-0.121	0.0409	-2.96	0.00306	**
absMnSal:OceanPacific	-0.125	0.0313	-3.98	6.86E-05	***
sdSal:propOxy	-0.0758	0.101	-0.752	0.452	NA
sdSal:OceanIndian	0.135	0.0948	1.42	0.155	NA
sdSal:OceanPacific	0.107	0.0689	1.56	0.119	NA
propOxy:OceanIndian	0.0159	0.124	0.128	0.898	NA
propOxy:OceanPacific	-0.153	0.105	-1.45	0.146	NA

Table 5 - Likelihood ratios and their significance for the different diversity models. Abbreviations: LR – likelihood ratio, p – p value, Signif – significance stars (*** < 0.001, 0.001 < ** < 0.01, 0.01 < * < 0.05, 0.05 < ˙ < 0.1).

**Environmental variable**	**Rarefied species richness**			**Simpson's Evenness**			**Average Community Age**			**Functional richness**		
	LR	p	Signif.	LR	p	Signif.	LR	p	Signif.	LR	p	Signif.
poly(mnSST, 3), 3	47.82285	6.69E-07	***	27.33984	0.0023	**	112.9867	1.33E-19	***	20.5209	0.024693	*
poly(mnSST, 3), 2	122.7387	8.85E-17	***	93.28151	1.97E-11	***	191.2587	5.98E-30	***	111.9182	8.77E-15	***
poly(mnSST, 3), 1	421.443	1.28E-70	***	131.9084	9.70E-15	***	220.5129	6.73E-31	***	390.652	2.15E-64	***
sdSST	13.17455	0.356482	NA	31.66994	0.001556	**	54.04245	2.69E-07	***	16.30474	0.177674	NA
I(MLD/10)	32.36301	0.001216	**	48.69955	2.36E-06	***	12.62936	0.396546	NA	21.61403	0.04208	*
I(depth10deg/100)	33.59469	0.000781	***	15.60149	0.210177	NA	15.85537	0.19795	NA	26.13081	0.010286	*
mn.logProd	47.41127	3.96E-06	***	34.23465	0.000619	***	25.39793	0.013046	*	45.10189	9.90E-06	***
absMnSal	44.33081	1.34E-05	***	14.90766	0.246525	NA	18.11133	0.112352	NA	42.63672	2.60E-05	***
sdSal	40.62945	5.65E-05	***	26.5183	0.009059	**	26.08289	0.010448	*	28.09003	0.005367	**
propOxy	28.11141	0.005328	**	22.73265	0.030083	*	25.36775	0.013173	*	11.66427	0.473005	NA
Ocean	79.40847	2.03E-08	***	53.15116	0.000216	***	33.65337	0.053255	.	68.07843	1.32E-06	***
dCarbIon	7.799426	0.005226	**	0.908158	0.340604	NA	17.18252	3.40E-05	***	0.015733	0.900181	NA
