# Supplementary material for: Environmental Predictors of Diversity in Recent Planktonic Foraminifera as Recorded in Marine Sediments
Source: PLoS One. 2016 Nov 16;11(11):e0165522. doi: 10.1371/journal.pone.0165522 (PMC5112986; doi:10.1371/journal.pone.0165522)
Supplement: S1 Table — Columns indicate the names in the MARGO dataset, the species used in this analysis and comments justifying the reasons. Coloured rows indicate definitions which were grouped. (PDF) [file pone.0165522.s008.pdf]

Supporting Table 1 - Species list used in this analysis. Columns indicate the names in the MARGO dataset, the species used in this analysis and comments justifying the reasons. Coloured rows indicate definitions which were grouped.

Species in MARGO	Species in this analysis	Comments
Beela digitata	*Beella digitata*	-
Globigerina bulloides	*G. bulloides*	-
Globigerina falconensis	*G. falconensis*	-
Globigerinella adamsi	*G. adamsi*	-
Globigerinella calida	*G. calida*	-
Globigerinella siphonifera (=aequilateralis)	*G. siphonifera*	
Globigerinoides conglobatus	*G. conglobatus*	-
Globigerinoides ruber (pink)<br>***Globigerinoides ruber (white)***<br>***Globigerinoides ruber total***	*G. ruber*	Don't split these as. The genetic data (Aurahs et al, 2011, MarMicro) highlights *G. ruber* (pink) is a separate genetic species from *G. ruber* (white). However *G. ruber* (white) also contains two genetic species, *G.ruber* and *G. elongatus*, which are more distinct than pink and white *G. ruber*. Given these latter two haven't been separated in the database, we are conservative in considering all these as the same morphological species.
Globigerinoides sacculifer with sac<br>***Globigerinoides sacculifer w/o sac***<br>***Globigerinoides sacculifer total***	*G. sacculifer*	These are not recognised as separate species (André et al, 2013, PalBio) so they were merged.
Globorotalia inflata	*G. inflata*	
Globoquadrina conglomerata	*G. conglomerata*	
Globorotalia menardii	*M. menardii*	
Globorotalia tumida	*G. tumida*	
Globorotalia menardii flexuosa	*G. flexuosa*	
Globorotalia menardii + tumida		The small number of sites (130) that had merged *G. menardii* and *G. tumida* were excluded from this analysis.
Globorotaloides hexagonus	*G. hexagonus*	
Globoturborotalita rubescens	*G. rubescens*	
Globoturborotalita tenella	*G. tenella*	
Globorotalia hirsuta	*H. hirsuta*	
Globorotalia scitula	*H. scitula*	

Globorotalia theyeri	*H. theyeri*	
Neogloboquadrina pachyderma L	*N. pachyderma*	
Neogloboquadrina pachyderma R	*N. incompta*	
P/D integrade + Neogloboquadrina pachyderma R		Following Morey et al (2005, QSR) where this column differed from *N. pachyderma* R, the extra individuals were added to *N. dutertrei*
Neogloboquadrina dutertrei	*N. dutertrei*	
Orbulina universa	*O. universa*	
Pulleniatina obliquiloculata	*P. obliquiloculata*	
Sphaeroidinella dehiscens	*S. dehiscens*	
Berggrenia pumilio + T. humilis		As this is not a species level identification it was excluded.
Turborotalita humilis	*T. humilis*	
Turborotalita quinqueloba	*T. quinqueloba*	
Globorotalia truncatulinoides L		Sinistral and dextral forms were not split in the Atlantic. There is also no clear genetic evidence that these are separate species (Ujiié et al., 2010, MarMicro). Therefore these two forms were not split.
Globorotalia truncatulinoides R		
Globorotalia truncatulinoides	*T. truncatulinoides*	
Globorotalia crassaformis	*T. crassaformis*	
Globorotalia crassula	*T. crassula*	This species is extinct, so should not be present in the analysis. Sites where this species was found were excluded from the analysis.
Candeina nitida	Micro	We did not include microperforate species in our study
Dentagloborotalia anfracta	Micro	
Globigerinita glutinata	Micro	
Globigerinita uvula	Micro	
Tenuitella iota	Micro	
Hastigerina pelagica	Micro	
